# Direct electrosynthesis of 52% concentrated CO on silver’s twin boundary

**DOI:** 10.1038/s41467-021-22428-1

**Published:** 2021-04-09

**Authors:** Can Tang, Peng Gong, Taishi Xiao, Zhengzong Sun

**Affiliations:** 1 Department of Chemistry and Shanghai Key Laboratory of Molecular Catalysis and Innovative Materials, Fudan University, Shanghai, P. R. China; 2 School of Microelectronics and State Key Laboratory of ASIC and System, Fudan University, Shanghai, P. R. China

**Keywords:** Electrocatalysis, Electrocatalysis, Energy, Carbon capture and storage

## Abstract

The gaseous product concentration in direct electrochemical CO_2_ reduction is usually hurdled by the electrode’s Faradaic efficiency, current density, and inevitable mixing with the unreacted CO_2_. A concentrated gaseous product with high purity will greatly lower the barrier for large-scale CO_2_ fixation and follow-up industrial usage. Here, we developed a pneumatic trough setup to collect the CO_2_ reduction product from a precisely engineered nanotwinned electrocatalyst, without using ion-exchange membrane. The silver catalyst’s twin boundary density can be tuned from 0.3 to 1.5 × 10^4^ cm^−1^. With the lengthy and winding twin boundaries, this catalyst exhibits a Faradaic efficiency up to 92% at −1.0 V and a turnover frequency of 127 s^−1^ in converting CO_2_ to CO. Through a tandem electrochemical-CVD system, we successfully produced CO with a volume percentage of up to 52%, and further transformed it into single layer graphene film.

## Abstract (web summary)

Isolating purified electrosynthesis product is a major challenge in electrochemical carbon dioxide reduction. Here, the authors report a nanotwinned silver electrocatalyst and a pneumatic-trough cell system to produce a 52% concentrated CO, which is further utilized as a carbon feedstock for graphene production.

## Introduction

Efficient catalyst design and complex product synthesis are two major challenges in electrochemical CO_2_ reduction reaction (CO_2_RR) toward industrial application. Defects engineering is one of the most effective strategies to promote catalyst’s performance, for defects have highly unsaturated coordinate-number and special electronic structure^1–3^. By introducing defects like doped atoms^4,5^, edges^6^, interphase boundaries^7,8^, grain boundaries^9^, and twin boundaries^10^ (TBs) in catalysts such as copper or silver, they tend to exhibit an outstanding activity with specific selectivity. Among those defects, TB, a mirror plane dividing the crystal lattice into two symmetric parts, shows many intriguing properties for its unique atomic arrangement^11,12^. Beneficial from the low interfacial energy, TB is one of the most stable defects in typical face-centered cubic (fcc) metals. Highly dense TBs have not only made copper highly conductive and mechanically stronger, but also more efficient to convert CO_2_ into CH_4_^10–12^. Different from Cu, metal silver can selectively transform CO_2_ into CO, owing to its low adsorption energy of CO and high overpotential for hydrogen evolution reaction (HER)^13,14^. The fivefold twinned silver nanowires have been reported to be a prominent catalyst in CO_2_RR^15^, and recently study suggests that TB in this kind of silver nanowires shows two orders higher activity than (100) facet of silver nanocubes^16^. However, the exact catalytic contribution of Ag’s TB in nanocrystal has been mixed with other factors such as strain, size-effect, and capping agent. Therefore, it is still challenging to reveal the TB’s catalytic contribution under a well-defined condition.

Fabricating highly dense TBs on a plane electrode can eliminate the unwanted factors in nano-sized catalysts^10^. Nanotwinned Ag (Nt-Ag) can be obtained through sputtering Ag on special substrates like single-crystalline faceted silicon^17^, which requires premium instruments and high-quality substrates, limiting its industrial application. Pulsed electrodeposition has been widely applied to synthesize nanotwinned copper^11,12^, but its success on silver has not been achieved yet.

On the other hand, fixing carbon from CO_2_ and converting them into high value-added products such as medical molecules or electronic-grade graphene is one of the ultimate goals in CO_2_ reduction. It has been reported that graphene sheets with many defects can be produced from CO_2_ in molten salt^18^. In mild aqueous conditions, C_5+_ products have never been directly synthesized using CO_2_ as the only carbon source, due to the high thermodynamic stability of CO_2_. An alternative way for CO_2_ conversion is a “two-step” strategy, where CO_2_ firstly transforms into CO and CO subsequently evolves in the next chemical reaction into other products^19,20^. Although it has been regarded as one of the most promising ways for complex product synthesis, unfortunately, conventional H-type cells can produce CO with a volume fraction level of ~0.01–2%^21–30^. This is because the CO product is always mixed with the original stream of CO_2_ in this system. With introducing a three-phase interface, a gas-diffusion electrode (GDE) can largely enhance the CO yield and lift the CO concentration to several tens of percent^21–23,31–39^. However, with the sophisticated membrane system, the efficiency of GDE relies on meticulous parameter control such as the thickness and hydrophobicity of functional layers. The ion-change membrane also hinders the mass transfer between anolyte and catholyte and depletes the electrolyte like bicarbonate in some certain setup. In addition, high-efficient GDEs are mainly made of particle-like samples as their catalytic layer. As for plate electrodes like metal foils, which are more common in industrial electrosynthesis, it still cannot be fabricated into a GDE. Recently, pure carbonate (or bicarbonate) reduction was reported as an alternative way for CO_2_ utilization^40–42^. Assisted by a bipolar membrane, CO_2_ in situ released from carbonate (or bicarbonate) can be further reduced to CO. Without additional gaseous CO_2_ complementing, the product concentration is able to maintain a high level, but the Faradaic efficiency (FE) is also limited by the insufficient CO_2_ supplement.

Here, we developed a facile way to synthesize Nt-Ag with controllable TB density, which can serve as a high-performance CO_2_RR catalyst. With a new CO_2_ conversion system based on a pneumatic trough, ultrahigh-concentration CO was produced without using an ion-exchange membrane. By channeling the reduced CO stream into a chemical vapor deposition (CVD) system, we could finally convert the carbon atoms from CO_2_ into highly value-added single-layer graphene with extraordinary high quality.

## Results and discussion

**Fig. 1:**
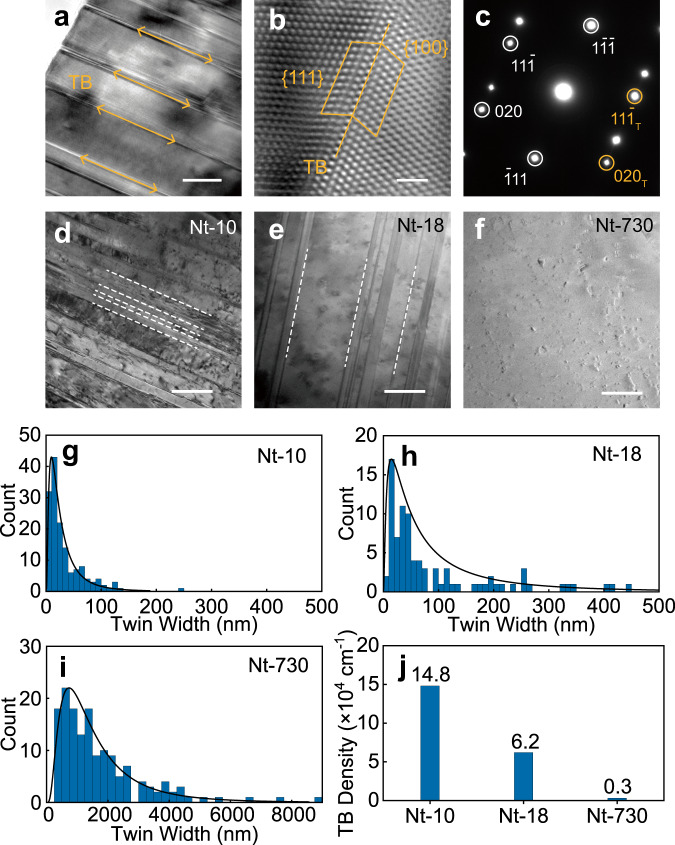
Silver catalysts with tunable TB density. **a** TEM image of parallel TBs (scale bar: 10 nm). TB represents twin boundary. **b** The atomic arrangement of a representative TB in FFT-treated HR-TEM (scale bar: 1 nm). The two marked rhomboids represent the Burgers circuits. **c** The typical SAED pattern of silver’s TB. The white and yellow circles label diffraction spots of matrix and twin, respectively. **d**–**f** TEM images of Nt-10, Nt-18, and Nt-730, respectively (scale bar: 100 nm). The white dashed lines mark TBs. **g**–**i** The twin width distribution of Nt-10, Nt-18, and Nt-730, respectively. **j** The statistical histogram of TB density.

The pulsed electrochemical deposition was introduced to synthesize highly dense TBs. Under  a pulsed current density of 1 A cm^−2^, the nanotwinned catalyst with the most probable twin width of 10 nm (Nt-10) was prepared. Its TB structure was further characterized with high-resolution electronic transmission microscopy (HRTEM) and selected area electron diffraction (SAED). Under TEM observation (Fig. 1a), there are many highly dense lines, which were recognized as silver {111} facet, parallelly emerging on Ag crystal. The lattices at both sides of the line (Fig. 1b) symmetrically arranged, showing a typical TB structure. The SAED pattern (Fig. 1c) shows two sets of diffraction spots symmetrical about a common {111} plane, further confirming a representative TB structure.

In order to control the TB density, we annealed Nt-10 at 300 and 800 °C for 2 h to obtain Nt-18 and Nt-730, and then measured their TB width and TB density with statistic methods from randomly selected TEM images (Supplementary Fig. 1). As shown in Fig. 1d–f, after annealing, the TB density remarkably decreases. The twin widths of Nt-10 mainly concentrate on ~10 nm and its corresponding TB density was measured as ~1.48 × 10^5^ cm^−1^ (Fig. 1g, j). In contrast to Nt-10, most TB widths in Nt-18 shift to ~18 nm (Fig. 1h), and the TB width distribution dispersion gets larger, giving rise to a lower TB density of 6.2 × 10^4^ cm^−1^. After annealing at 800 °C, only ~2% TBs remain in Nt-730. The twin widths in Nt-730 grew up with a distribution from hundreds of nanometers to several microns and the most probable twin width expands to 730 nm (Fig. 1i).

In some cases, high-temperature annealing can change the catalyst surface morphology and facet orientation, which leads to unexpected variation in its catalytic performance. To investigate our samples’ surfaces change, we carried out scanning electron microscopy (SEM) observation and surface roughness factor measurement. After annealed at 300 and 800 °C, the macroscopical surface morphology barely changes (Supplementary Fig. 2). Annealing could rearrange the atoms of the surface at an atomic level, yet it is not enough to reshape the bulked Ag’s macroscopical morphology. The roughness factors measured by cyclic voltammetry (CV) of Nt-10, Nt-18, and Nt-730 are 8.8, 8.2, and 8.4, respectively (Supplementary Fig. 3 and Supplementary Table 1), further confirming that the surface morphology of all samples is similar. Facet orientation was evaluated with X-ray diffraction (XRD). For Nt-10, besides low-index (111) and (200) planes were detected at the 2*θ* of 38.4° and 44.5° (Supplementary Fig. 4), the high-index (220) and (311) which usually have higher catalytic activity were also found at 64.6° and 77.6°. To our surprise, even after annealing at 800 °C, the high-index facets still existed. The peak ratio of the high-index facet only changed slightly, although the results represent the bulk information, which can not be extrapolated to the surface facet change. In brief, by using pulsed electrodeposition and annealing methods, we obtained a series of bulked Ag samples with controllable TB density and similar surface circumstances, which were further used as a defect model to study CO_2_RR on TBs.

**Fig. 2:**
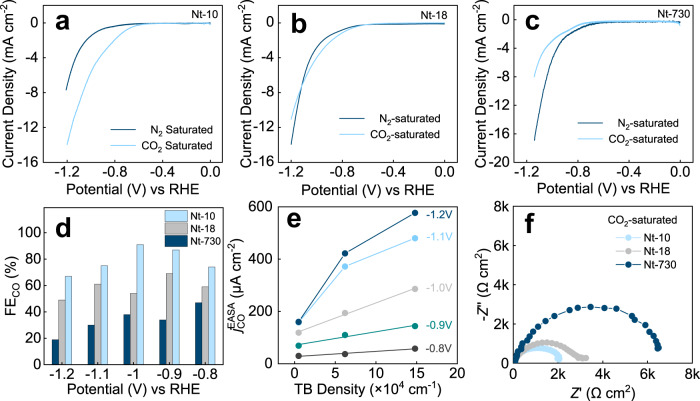
The CO production on the silver’s TBs. LSV curves obtained on **a** Nt-10, **b** Nt-18, and **c** Nt-730 in N_2_-saturated and CO_2_-saturated electrolyte. **d** The FE_CO_ under different applied cathode potentials in CO_2_-saturated electrolyte. **e** The correlation between $j_{{\mathrm{CO}}}^{{\mathrm{EASA}}}$ and TB density at different potentials. **f** Nyquist plots of Nt-10, Nt-18, and Nt-730 at −0.7 V in CO_2_-saturated solution. All electrochemical measurements were carried out in a 0.5 M KHCO_3_ solution.

The catalytic performance of CO_2_RR on TBs was preliminarily investigated by linear scanning voltammetry (LSV) in CO_2_-saturated and N_2_-saturated solutions. As shown in Fig. 2a–c, on Nt-10, the current density (*j*) in CO_2_-saturated solution is larger than that in N_2_-saturated solution after −0.6 V, suggesting a preference of CO_2_RR over its competing reaction, namely the hydrogen evolution reaction. However, as TB density decreases, the *j* in the N_2_-saturated solution starts to rise and the counterpart in CO_2_-saturated solution gradually declines. Eventually, the *j* in N_2_-saturated solution on Nt-730 surpasses that in CO_2_-saturated solution, indicating the HER becomes dominated when TBs are deficient. A similar trend was also observed in their Nyquist plots (Supplementary Fig. 5 and Supplementary Table 2). For Nt-10, the charge-transfer resistance (*R*_ct_) in CO_2_-saturated solution (~2.0 kΩ cm^2^) is smaller than that in N_2_-saturated solution (~4.9 kΩ cm^2^). However, for Nt-730, this rank of value is inverse, proving that electrons on TBs could more easily transfer to CO_2_ rather than H^+^.

The precise gas and liquid products were further analyzed with gas chromatography (GC) and ^1^H nuclear magnetic resonance (NMR), respectively, (Supplementary Fig. 6). The GC spectrum shows that CO and H_2_ are the only gaseous products for all samples, while no any liquid products were detected. For Nt-10, the CO started to produce at a low potential of −0.8 V with a FE_CO_ of 74% in CO_2_-saturated 0.5 M KHCO_3_ solution and reached its maximum FE_CO_ of 92% at −1.0 V (Fig. 2d). With TB density declines, the FE_CO_ of Nt-18 and Nt-730 gradually decreased to 54 and 38% at −1.0 V, which manifests TB enhances the selectivity of CO production.

Our previous work reported a method to estimate the intrinsic activity of copper TB by correlating its activity with the corresponding density^10^. Using the same method, we separated intrinsic partial current densities of CO (*j*_CO_) on TB from that on plane atoms. In order to eliminate the effect brought by the difference of surface roughness, we used electrochemical active surface area (EASA) to normalize *j*_CO_ ($j_{{\mathrm{CO}}}^{{\mathrm{EASA}}}$) to represent the activity of CO evolution. As shown in Fig. 2e, $j_{{\mathrm{CO}}}^{{\mathrm{EASA}}}$ displays a linear relation with TB density from −0.8 to −1.0 V. The values of slope and intercept represent the intrinsic *j*_CO_ on TB and plane, respectively. The calculated results are summarized in Supplementary Fig. 7. Notably, at −1.0 V, the intrinsic *j*_CO_ on TB is ~33 mA cm^−2^, corresponding to the turnover frequency (TOF) of 127 s^−1^, which is believed to be the highest value in electrochemical CO evolution from CO_2_ (Supplementary Fig. 8 and Supplementary Table 3). By contrast, the *j*_CO_ on a plane is only ~0.1 mA cm^−2^ at the same condition, corresponding to the TOF of 0.39 s^−1^, ~0.3% of the activity on TB atoms. At more negative potentials, such linear relation was not observed, for the mass-transfer process becomes dominated on TB-rich samples.

The pulsed electrochemical deposition allows us to fabricate TBs on diverse substrates such as inert metals and carbon materials (Supplementary Fig. 9). The Nt-Ag deposited on different substrates remains similarly high catalytic performance (Supplementary Fig. 10), which endows Nt-Ag with universal application in future. As TB is one of the most stable defects in fcc metals, the Nt-10 can endure long-time reaction with nearly no performance decline in 32 h (Supplementary Fig. 11). The SEM images and XRD patterns before and after reaction did not show a significant change in Nt-10’s morphology and facet orientation (Supplementary Fig. 12 and Supplementary Fig. 13). More detailed structural information was given by TEM observation (Supplementary Fig. 14). After a 12-h reaction, TB still maintained its density with a negligible change in twin width. For other active centers, we observed that dislocations changed both in position and atomic arrangement and the facets were slightly contaminated, further displaying the prominent stability of TBs over other defects.

The superior catalytic performance of Nt-10 is attributed to that TBs lower the barrier of the charge-transfer process from the electrode to CO_2_ and dynamically weaken the activity of competing HER. In CO_2_-saturated electrolyte, Nt-730 has a high *R*_ct_ of ~6.3 kΩ cm^2^ (Fig. 2f and Supplementary Table 2), while highly dense TBs in Nt-10 remarkably reduce *R*_ct_ to ~2.0 kΩ cm^2^, fastening the CO evolution on silver. In contrast, the competing HER on TB was largely suppressed. At all potentials, both FE_H2_ and $j_{{\mathrm{H2}}}^{{\mathrm{EASA}}}$ show a conspicuous decrease with TB density rising (Supplementary Fig. 15). Further chemically dynamic research using Tafel plots and Nyquist plots in N_2_-saturated electrolyte were carried out to investigate about the competition with HER. The Tafel slopes on Nt-10 and Nt-730 were measured as 301 and 276 mV dec^−1^ (Supplementary Fig. 16), suggesting that TB dynamically deactivates the HER on silver. The corresponding Nyquist plots (Supplementary Fig. 17) also show a smaller *R*_ct_ on Nt-730 than Nt-10’s, which confirms HER has a higher dynamic barrier of electron-transfer from TB.

**Fig. 3:**
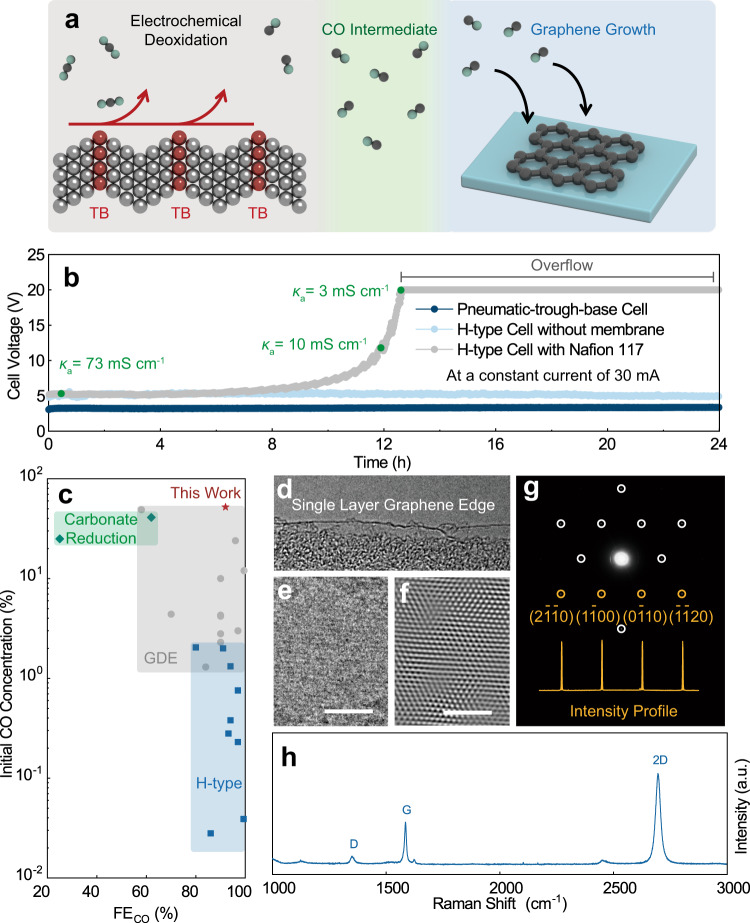
Tandem system for graphene synthesis. **a** The schematic diagram of “two-step” synthesis of graphene from CO_2_. **b** Cell voltages at a constant current density of 30 mA cm^−2^ in pneumatic-trough-based cell and H-type cell with/without Nafion 117 membrane. The area of the electrode is ~1 cm^2^. The “overflow” represents the applied voltage reaches the maximum of our electrochemical workstation (20 V). The *κ*_a_ represents the electric conductivity of anolyte. **c** The literature summary of FE_CO_ and initial CO concentration^21–38,40,41^. The red, green, gray, and blue spots represent the results of this work, carbonate reduction systems, GDE systems, and H-type cell systems, respectively. **d** The TEM image of graphene film edge (scale bar: 10 nm) The **e** raw and **f** FFT-treated HRTEM images of graphene film lattice (scale bar: 2 nm). **g** The SAED pattern of graphene film and the intensity profile of diffraction spots along (2$\overline 1 \overline 1$0), (1$\overline 1$00), (0$\overline 1$10), and ($\overline 1 \overline 1$20) facet. **h** Raman spectrum of graphene film.

With the superior catalytic performance on Nt-10, we designed a tandem system based on a pneumatic trough to collect CO product and further transform it into graphene film (Fig. 3a). In conventional H-type cells or GDE, CO_2_ and gas products share the same outlet, and the products are therefore diluted by CO_2_. In our system, the product is separated from the inlet of CO_2_ with a pneumatic trough (Supplementary Fig. 18). When CO_2_ was bubbled into the electrolyte, the dissolved CO_2_ can diffuse to the surface of the electrode while undissolved gas was expelled outside the pneumatic trough. Therefore, the CO product is isolated by the pneumatic trough from CO_2_ feedstock and the anodic products without using premium ion-exchange membranes. Compared with the GDE structure, this system is suitable for more types of solid materials, including metal plates and particles on appropriate supports. Moreover, the absence of an ion-exchange membrane simplifies the cell structure and facilitates the mass exchange between anolyte and catholyte. We performed the chronopotentiometry at a constant current of 30 mA in pneumatic-trough-based cell and commercial H-type cell with/without Nafion 117 membrane for 24 h (Fig. 3b). The initial cell voltage of our system is ~3.3 V, slightly lower than that of the commercial H-type cell, which is mainly attributed to the lower ohmic resistance originated from different electrolyzers’ shapes (Supplementary Fig. 19). We observed that both the pneumatic-trough-based cell and the H-type cell without using Nafion 117 membrane maintained stable cell voltages, while in a Nafion 117 membrane system, the cell voltage continuously climbing after 8 h. In a detailed pH and conductivity study (Supplementary Fig. 20), the possible mechanism can be attributed to inefficient ion exchange, marked with a continuous depletion of KHCO_3_ in the anolyte, and a continuous accumulation of KHCO_3_ in the catholyte. By choosing different electrolytes at both sides, such as using H_2_SO_4_ as the anolyte and KHCO_3_ as the catholyte, the cell voltage can keep stable for a longer time, despite that a small amount of CO_2_ may form at the interface between Nafion membrane and KHCO_3_ catholyte (Supplementary Fig. 21). Overall, the low ohmic resistance and higher cell voltage stability of pneumatic-trough-based cell lifts the energy conversion efficiency and benefits an energy conversion efficiency of up to ~53% on Nt-10 (Supplementary Fig. 22).

More importantly, the CO product in our system would not be directly diluted by the CO_2_ feedstock. The theoretical ceiling of CO concentration in this system equals to catalyst’s FE_CO_. However, under real electrochemical circumstances, the CO_2_ will escape from the CO_2_-saturated electrolyte. The escape rate can be influenced by the temperature, electrolyzer shape, and roughness of the electrode^43^. Under our most optimal condition, the initial concentration of CO can reach up to 52% (Supplementary Fig. 23), higher than the CO concentration in most GDE systems (Fig. 3c and Supplementary Table 4). The high concentration of CO makes it possible to be applied in industrial processes. With simple gas purification, we pumped CO product into tube furnace for graphene growth. The detailed growth conditions were described in the Supplementary Information and Supplementary Fig. 24.

Graphene is a valuable carbon material with fascinating performance in electronic devices^44^. Many efforts have been devoted to converting CO_2_ to graphene, yet high-quality graphene film has not been achieved^45,46^. The major obstacle is the two oxygen atoms in CO_2_. Although it has been reported a moderate amount of oxygen is beneficial to high-quality graphene growth^47,48^, surplus oxygen in the CO_2_ molecules, however, can etch graphene^49^. With partially deoxidize CO_2_ into CO, CO can transform into a continuous graphene film (Supplementary Fig. 25). The TEM image of the film’s edge indicates most area of the film is single layer (Fig. 3d), and its lattice structure under HRTEM observation is perfect with few defects (Fig. 3e, f). Its corresponding SAED spots also exhibit a typical single-layer graphene pattern (Fig. 3g). Particularly, the diffraction intensities of (2$\overline 1 \overline 1$0), (1$\overline 1$00), (0$\overline 1$10), and ($\overline 1 \overline 1$20) are about the same, consistent with the feature of single-layer graphene^50^. Further Raman spectrum analysis (Fig. 3h) shows an *I*_G_/*I*_2D_ of 0.45 and a full width at half maximum (FWHM) of 25 cm^−1^ for 2D peak, corresponding to single-layer structure^51,52^. The low *I*_D_ suggests the film is with few defects.

By contrast, we pumped pure CO_2_ into our system under similar conditions, but no graphene film formed. Instead, CO_2_ transforms into snow-flake-like graphene fragments with an average size of ~16 μm (Supplementary Fig. 26). The discontinuity of graphene fragments is mainly due to the etching of CO_2_. Its TEM observation (Supplementary Fig. 27) indicates the layer number of most graphene is more than 7. Raman spectrum (Supplementary Fig. 28) also shows that *I*_G_/*I*_2D_ at the most regions is higher than 2.75 with a very large FWHM of the 2D peak of ~60 cm^−1^, manifesting a much thicker graphene layer.

In conclusion, we developed a method to synthesize TBs on Ag, with a TB density of 1.5 × 10^5^ cm^−1^. Powered by these TBs, the electrocatalyst can easily transfer electron to CO_2_ and surpass the competing HER. With a tandem two-step system, the direct CO product from CO_2_RR can reach an industrial concentration without using ion-exchange membrane, and successfully transformed into high quality single-layer graphene. This work further fulfills our understanding of CO_2_RR with controllable TB defects and boosts the development of electrochemical CO_2_RR toward industrial production.

## Methods

### Silver catalyst preparation

The Nt-10 was deposited on a piece of graphite paper or other substrates using current-pulse mode in a two-electrode system, with an anode of silver foil. The electrolyte is a 1 M AgNO_3_ solution. The on-time and off-time of the pulsed current were set as 20 and 2000 ms, respectively, with the peak current density of 1 A cm^−2^. After deposition, the as-prepared Ag was stripped from the graphite paper.

The Nt-18 and Nt-730 were synthesized by annealing the Nt-10 at 300 and 800 °C, respectively, in the H_2_ atmosphere for 2 h.

### Graphene growth

Graphene film was synthesized with the CVD method on copper foil. First, a piece of copper foil with a thickness of 30 μm was electrochemically polished in 85wt.% H_3_PO_4_ solution for ~1 min. The counter electrode is also copper foil with twenty times larger area. The applied voltage is 5 V. The as-prepared copper foil was then put in tube furnace for graphene growth. The tandem graphene growth system is composed of three functional parts (Supplementary Fig. 22)—gas purification part, CO activation part and graphene growth part. The gas purification part contains three purification tubes filled with NaOH/asbestos, dried silica gel and MnO, respectively, in order to remove unfavored CO_2_, water and O_2_. The CO activation part is 6 g of commercial Ni/Al_2_O_3_ catalyst (Sichuan Shutai Co. Ltd.), which can transform CO into hydrocarbon intermediates. The graphene growth part is a conventional CVD tube furnace. Before graphene growth, the copper foil was annealing in air atmosphere (25 mttor) at 1060 °C for 10 s, then H_2_ (50 sccm, ~8000 Pa) as the reductant was flowed into quartz tube. Subsequently, 300 mL of CO product was pumped into the tandem system. With adjusting the flow rate of CO product, the total pressure was controlled at ~8400 Pa for at least 5 min. Graphene fragment using pure CO_2_ was synthesized in the similar way, but NaOH/asbestos and MnO was removed for they can adsorb CO_2_.

### Characterization of structure

The structure characterization condition is similar to our previous work^10^. XRD was carried out with a Bruker AXS D8 Advance X-ray Powder Diffractometer operated at 1600 W power (40 kV, 40 mA) using Cu Kα radiation. Transmission electron microscopy (TEM) were performed on an HT7700 Exalens operated at 120 kV. High-resolution TEM observation was carried out with a Tecnai G2 F20 S-Twin microscopy operated at 200 kV. Scanning electron microscopy (SEM) was carried out on a Phenom Prox operated at 15 kV. Raman spectrum was performed with a Jobin Yvon Lab RAM HR800 equipped with a 514 nm laser.

### EASA measurement

A CV from −0.1 to 0.1 V (vs. RHE, except as otherwise noted), was carried out with the different scan rates of 40, 60, 80, 100, and 120 mV s^−1^ in a CO_2_-saturated 0.5 M KHCO_3_ solution at room temperature. We correlated the charging current density at 0.0 V with its corresponding scan rate. The double-layer capacitance (*C*_dl_) is the slope of the fitted line.

### LSV measurement

The LSV measurement was carried out in N_2_-saturated or CO_2_-saturated 0.5 M KHCO_3_ solution from 0 to −1.2 V at a scan rate of 50 mV s^−1^.

### EIS measurement

The EIS measurement was carried out at −0.7 V with a frequency from 0.1 Hz to 100 kHz, and an amplitude of 5 mV. The measured impedances were normalized by multiplying the EASA.

### Catalytic evaluation of CO_2_ reduction

CO_2_ reduction was carried out in 0.5 M KHCO_3_ solution. The counter electrode and reference electrode are a 3 × 4 cm^2^ of IrO_2_ mesh electrode and the Ag/AgCl (3 M KCl solution), respectively. The potential vs. RHE was converted from the potential vs. Ag/AgCl by the following equation:

1 $$E_{{\mathrm{RHE}}} = E_{{\mathrm{Ag/AgCl}}}+ {\mathrm{ 0}}{\mathrm{.197 + 0}}{\mathrm{.059pH}}$$

CO_2_ was bubbled into electrolyte before the electrochemical measurement to reach a saturated concentration. After reaction at a set potential, we recorded the total amount of electrical charge (*Q*) and collected the cathodic gas in a certain volume (*V*_g_) with a home-built electrochemical cell (Supplementary Fig. 18). The Faradaic efficiency of the gas products (FE_g_) was analyzed with the GC equipped with a thermal conductivity detector (TCD) and a hydrogen flame ionization detector (FID). The collected gas products were injected into GC to obtain their concentration (*C*_g_). The FE_g_ was calculated as the following equation:

2 $${\mathrm{FE}}_g = \frac{{{{zPF}} \times V_g \times C_g}}{{{\mathrm{RTQ}}}}$$

where *R*, *F*, *z*, *P*, and *T* represent gas constant, Faradaic constant, the number of transferred charges, environment pressure, and temperature, respectively.

The liquid products were detected with ^1^H NMR recorded by a Bruker Avance III HD (500 MHz). We added electrolyte into the cathodic part of an H-type cell, which is separated by a Nafion-117 ion-exchange membrane. After the reaction finished, the electrolyte was mixed up with D_2_O and DMSO and the liquid products were further detected by ^1^H NMR.

## Supplementary information

Supplementary Information

## Data Availability

The authors declare that the data supporting the findings of this study are available within the article and the corresponding Supporting Information file. All other relevant source data are available from the corresponding author upon reasonable request.

## References

[1] Wang Y, Han P, Lv X, Zhang L, Zheng G (2018). Defect and interface engineering for aqueous electrocatalytic CO_2_ reduction. Joule.

[2] Geng Z (2018). Oxygen vacancies in ZnO nanosheets enhance CO_2_ electrochemical reduction to CO. Angew. Chem. Int. Ed..

[3] Chen Z, Zhang X, Lu G (2015). Overpotential for CO_2_ electroreduction lowered on strained penta-twinned Cu nanowires. Chem. Sci..

[4] Sharma PP (2015). Nitrogen‐doped carbon nanotube arrays for high‐efficiency electrochemical reduction of CO_2_: on the understanding of defects, defect density, and selectivity. Angew. Chem. Int. Ed..

[5] Clark EL, Hahn C, Jaramillo TF, Bell AT (2017). Electrochemical CO_2_ reduction over compressively strained CuAg surface alloys with enhanced multi-carbon oxygenate selectivity. J. Am. Chem. Soc..

[6] Abbasi P (2016). Tailoring the edge structure of molybdenum disulfide toward electrocatalytic reduction of carbon dioxide. ACS Nano.

[7] Lee S, Park G, Lee J (2017). Importance of Ag–Cu biphasic boundaries for selective electrochemical reduction of CO_2_ to ethanol. ACS Catal..

[8] Morales-Guio CG (2018). Improved CO_2_ reduction activity towards C_2_+ alcohols on a tandem gold on copper electrocatalyst. Nat. Catal..

[9] Feng X, Jiang K, Fan S, Kanan MW (2015). Grain-boundary-dependent CO_2_ electroreduction activity. J. Am. Chem. Soc..

[10] Tang C (2020). CO_2_ reduction on copper’s twin boundary. ACS Catal..

[11] Lu K (2016). Stabilizing nanostructures in metals using grain and twin boundary architectures. Nat. Rev. Mater..

[12] Lu L, Shen Y, Chen X, Qian L, Lu K (2004). Ultrahigh strength and high electrical conductivity in copper. Science.

[13] Kuhl KP (2014). Electrocatalytic conversion of carbon dioxide to methane and methanol on transition metal surfaces. J. Am. Soc. Chem..

[14] Nørskov JK (2015). Trends in the exchange current for hydrogen evolution. J. Electrochem. Soc..

[15] Liu S (2018). Ultrathin 5-fold twinned sub-25 nm silver nanowires enable highly selective electroreduction of CO_2_ to CO. Nano Energy.

[16] Hu F (2020). Quantifying electrocatalytic reduction of CO_2_ on twin boundaries. Chemistry.

[17] Bufford D, Wang H, Zhang X (2011). High strength, epitaxial nanotwinned Ag films. Acta Mater..

[18] Hu L (2016). Direct conversion of greenhouse gas CO_2_ into graphene via molten salts electrolysis. ChemSusChem.

[19] Luc W (2019). Two-dimensional copper nanosheets for electrochemical reduction of carbon monoxide to acetate. Nat. Catal..

[20] Zhao C (2020). In situ topotactic transformation of an interstitial alloy for CO electroreduction. Adv. Mater.

[21] Jiang K (2018). Isolated Ni single atoms in graphene nanosheets for high-performance CO_2_ reduction. Energy Environ. Sci..

[22] Luo W, Zhang J, Li M, Züttel A (2019). Boosting CO production in electrocatalytic CO_2_ reduction on highly porous Zn catalysts. ACS Catal..

[23] Jiang K (2017). Transition-metal single atoms in a graphene shell as active centers for highly efficient artificial photosynthesis. Chemistry.

[24] Luo W (2018). Selective and stable electroreduction of CO_2_ to CO at the copper/indium interface. ACS Catal..

[25] Yang HB (2018). Atomically dispersed Ni (I) as the active site for electrochemical CO_2_ reduction. Nat. Energy.

[26] Wang X (2018). Regulation of coordination number over single Co sites: triggering the efficient electroreduction of CO_2_. Angew. Chem..

[27] Jiang K, Wang H, Cai W-B, Wang H (2017). Li electrochemical tuning of metal oxide for highly selective CO_2_ reduction. ACS Nano.

[28] Jiang X (2017). Boosting CO_2_ electroreduction over layered zeolitic imidazolate frameworks decorated with Ag_2_O nanoparticles. J. Mater. Chem. A.

[29] Gao Y (2019). Enhanced selectivity and activity for electrocatalytic reduction of CO_2_ to CO on an anodized Zn/carbon/Ag electrode. J. Mater. Chem. A.

[30] Pan Y (2018). Design of single-atom Co–N_5_ catalytic site: a robust electrocatalyst for CO_2_ reduction with nearly 100% CO selectivity and remarkable stability. J. Am. Chem. Soc..

[31] Zhang X (2020). Molecular engineering of dispersed nickel phthalocyanines on carbon nanotubes for selective CO_2_ reduction. Nat. Energy.

[32] Daiyan R (2020). Transforming active sites in nickel–nitrogen–carbon catalysts for efficient electrochemical CO_2_ reduction to CO. Nano Energy.

[33] Dinh C-T, García de Arquer FP, Sinton D, Sargent EH (2018). High rate, selective, and stable electroreduction of CO_2_ to CO in basic and neutral media. ACS Energy Lett..

[34] Haas T, Krause R, Weber R, Demler M, Schmid G (2018). Technical photosynthesis involving CO_2_ electrolysis and fermentation. Nat. Catal..

[35] Verma S (2017). Insights into the low overpotential electroreduction of CO_2_ to CO on a supported gold catalyst in an alkaline flow electrolyzer. ACS Energy Lett..

[36] Salvatore DA (2017). Electrolysis of gaseous CO_2_ to CO in a flow cell with a bipolar membrane. ACS Energy Lett..

[37] Romero Cuellar NS (2020). Two-step electrochemical reduction of CO_2_ towards multi-carbon products at high current densities. J. CO2 Util..

[38] Krause R (2020). Industrial application aspects of the electrochemical reduction of CO_2_ to CO in aqueous electrolyte. Chem. Ing. Tech..

[39] Gabardo CM (2019). Continuous carbon dioxide electroreduction to concentrated multi-carbon products using a membrane electrode assembly. Joule.

[40] Li YC (2019). CO_2_ electroreduction from carbonate electrolyte. ACS Energy Lett..

[41] Lees EW (2020). Electrodes designed for converting bicarbonate into CO. ACS Energy Lett..

[42] Li T (2019). Electrolytic conversion of bicarbonate into CO in a flow cell. Joule.

[43] Angulo A, Linde PVD, Gardeniers H, Modestino M, Rivas DF (2020). Influence of bubbles on the energy conversion efficiency of electrochemical reactors. Joule.

[44] Schwierz F (2010). Graphene transistors. Nat. Nanotechnol..

[45] Luo B (2013). Synthesis and morphology transformation of single-crystal graphene domains based on activated carbon dioxide by chemical vapor deposition. J. Mater. Chem. C.

[46] Molina-Jirón (2019). Direct conversion of CO_2_ to muti-layer graphene using Cu-Pd alloys. ChemSusChem.

[47] Liu B (2017). Towards the standardization of graphene growth through carbon depletion, refilling and nucleation. Carbon.

[48] Zhang J (2019). Large-area synthesis of super-clean graphene via selective etching of amorphous carbon with carbon dioxide. Angew. Chem. Int. Ed..

[49] Yang X (2017). Insight into CO_2_ etching behavior for efficiently nanosizing graphene. Adv. Mater. Interfaces.

[50] Meyer JC (2007). On the roughness of single-and bi-layer graphene membranes. Solid State Commun..

[51] Ferrari AC (2006). Raman spectrum of graphene and graphene layers. Phys. Rev. Lett..

[52] Wang YY (2008). Raman studies of monolayer graphene: the substrate effect. J. Phys. Chem. C.

