# Supplementary material for: Direct electrosynthesis of 52% concentrated CO on silver’s twin boundary
Source: Nat Commun. 2021 Apr 9;12:2139. doi: 10.1038/s41467-021-22428-1 (PMC8035331; doi:10.1038/s41467-021-22428-1)
Supplement: Supplementary file 1 — Supplementary Information [file 41467_2021_22428_MOESM1_ESM.pdf]

Supplementary Information

Direct Electrosynthesis of 52% Concentrated CO on Silver's Twin Boundary

Can Tang[1], Peng Gong[1], Taishi Xiao[1], Zhengzong Sun[1,2]*

[1]Department of Chemistry and Shanghai Key Laboratory of Molecular Catalysis and Innovative Materials, Fudan University, Shanghai 200433, P. R. China

[2]School of Microelectronics and State Key Laboratory of ASIC and System, Fudan University, Shanghai 200433, P. R. China

**Correspondence to: zhengzong_sun@fudan.edu.cn*

Content

Supplementary Figure 1. TB density calculation. (Page 4)

Supplementary Figure 2. Surface morphology of Nt-10 before and after annealing. (Page 5)

Supplementary Figure 3. Double-layer current density at different scan rates. (Page 6)

Supplementary Table 1. The C_{dl} and roughness factors of Nt-10, Nt-18 and Nt-730. (Page 6)

Supplementary Figure 4. Crystal facet characterization of silver samples. (Page 7)

Supplementary Figure 5. Electrochemical impedance measurements. (Page 8)

Supplementary Table 2. Fitted charge-transfer resistance (R_{ct}) of Nt-10, Nt-18 and Nt-730 in CO_2-saturated and N_2-saturated solution. (Page 8)

Supplementary Figure 6. Gaseous and liquid products detection after CO_2RR. (Page 9)

Supplementary Figure 7. Intrinsic electrocatalytic activity calculation. (Page 10)

Supplementary Figure 8. The TOF_{CO} and FE_{CO} reported in Literatures 1-17. (Page 12)

Supplementary Table 3. Literature summary about TOF_{CO} and FE_{CO}. (Page 13)

Supplementary Figure 9. Surface morphology of Nt-10 deposited on different substrates. (Page 14)

Supplementary Figure 10. The FE_{CO} of Nt-10 on different substrates. (Page 15)

Supplementary Figure 11. Electrocatalytic stability of Nt-10. (Page 16)

Supplementary Figure 12. Structural stability of Nt-10 in SEM observation. (Page 17)

Supplementary Figure 13. Structural stability of Nt-10 in XRD measurement. (Page 18)

Supplementary Figure 14. Structural stability of Nt-10 in TEM observation. (Page 19)

Supplementary Figure 15. Electrocatalytic performance of hydrogen evolution on silver catalysts. (Page 20)

Supplementary Figure 16. Tafel plots in N_2-saturated solution on Nt-10 and Nt-730. (Page 21)

Supplementary Figure 17. Nyquist plots in N_2-saturated solution on Nt-10 and Nt-730. (Page 22)

Supplementary Figure 18. Schematic diagram of pneumatic-trough-based electrochemical cell. (Page 23)

Supplementary Figure 19. Electrical impedance measurement of different systems. (Page 25)

Supplementary Figure 20. The schematic of ion migration in proton-exchange membrane (PEM) system and membrane-free system. (Page 26)

Supplementary Figure 21. Electrochemical measurement using sulfuric acid as anolyte in H-type cell. (Page 28)

Supplementary Figure 22. Energy efficiency measurement and calculation. (Page 29)

Supplementary Figure 23. CO concentration measurement. (Page 30)

Supplementary Table 4. Literature summary about FE_{CO} and initial CO concentration. (Page 31)

Supplementary Figure 24. Graphene growth system. (Page 32)

Supplementary Figure 25. Optical image of graphene film on SiO_2 substrate synthesized from CO product. (Page 33)

Supplementary Figure 26. Optical image of graphene fragment on SiO_2 substrate synthesized from pure CO_2. (Page 34)

Supplementary Figure 27. TEM images of graphene fragments from pure CO_2. (Page 35)

Supplementary Figure 28. Raman spectrum of graphene fragments synthesized from pure CO_2. (Page 36)

References. (Page 37)

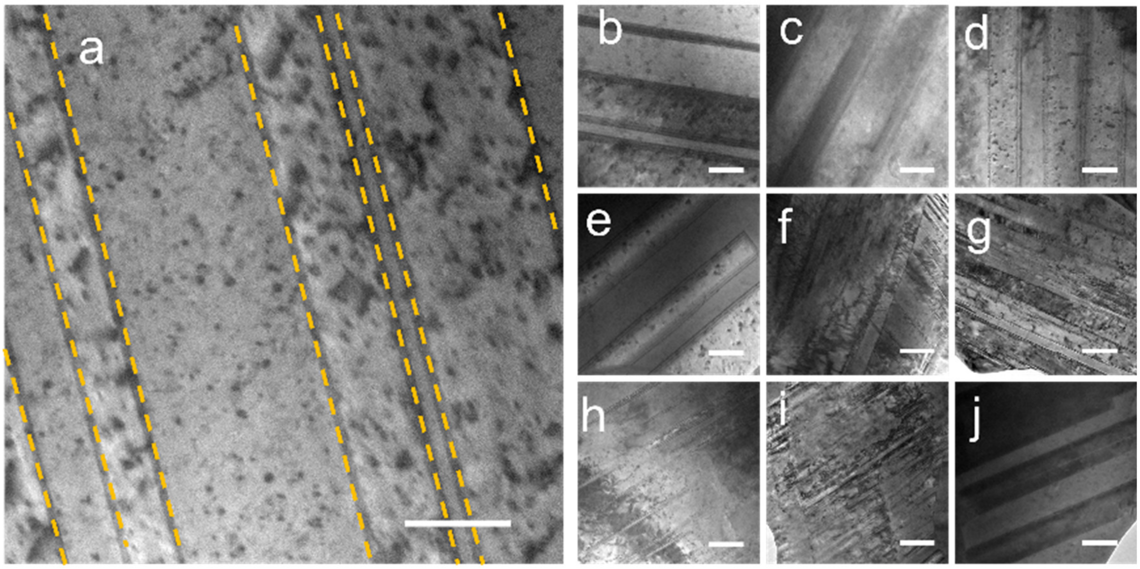

Supplementary Figure 1. TB density calculation. (**a**) The TEM image of Nt-10 as an example to illustrate how to calculate the TB density (The yellow dash lines mark the TBs.). (**b**)- (**j**) Other TEM images for the Nt-10 calculation. (scale bar: 100 nm)

The silver samples were thinned with a precise ion polishing system (PIPS II 695, GATAN). We randomly selected 10 regions under TEM observation, and then measured TBs' total length (L) and the area of the image (A) from the 10 images (Fig. S1a-1j**)**. The TB density can be estimated by the following equation:

$$\text{TB density}=\frac{L}{A} \quad (1)$$

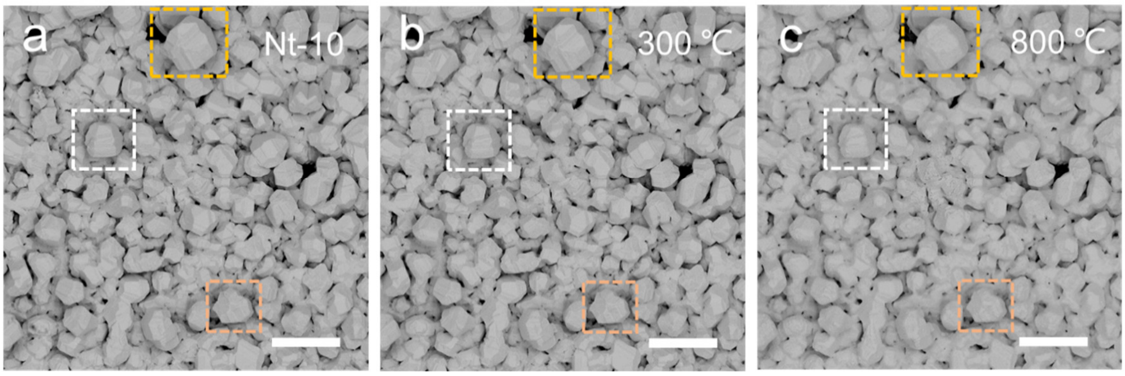

Supplementary Figure 2. Surface morphology of Nt-10 before and after annealing.

(**a**) Original SEM image of Nt-10. (**b**) SEM image of Nt-10 after annealing at 300 °C.

(**c**) SEM image of Nt-10 after annealing at 800 °C. (scale bar: 100 μm)

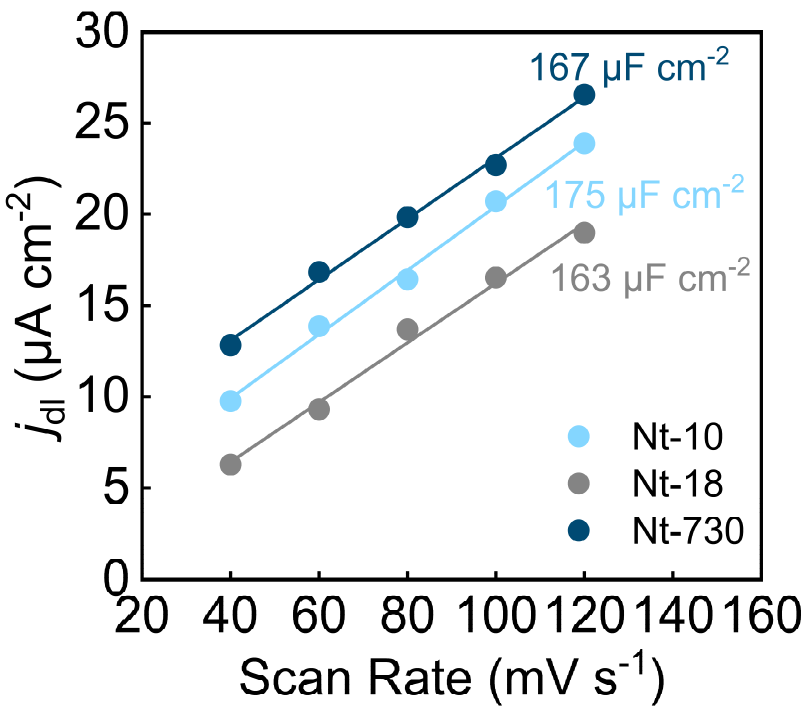

Supplementary Figure 3. Double-layer current density at different scan rates. The C_{dl} is calculated from the corresponding slope.

Supplementary Table 1. The C_{dl} and roughness factors of Nt-10, Nt-18 and Nt-730.

Samples	C_{dl} (μF cm^{-2})	Roughness Factor
Nt-10	175	8.8
Nt-18	163	8.2
Nt-730	167	8.4

The double-layered capacitance of a flat metal electrode is ~ 20 μF cm^{-2}. The roughness factor can be calculated by equation (4):

$$\text{Roughness Factor}=\frac{C_{dl}}{20\ \mu\text{F cm}^{-2}} \quad (2)$$

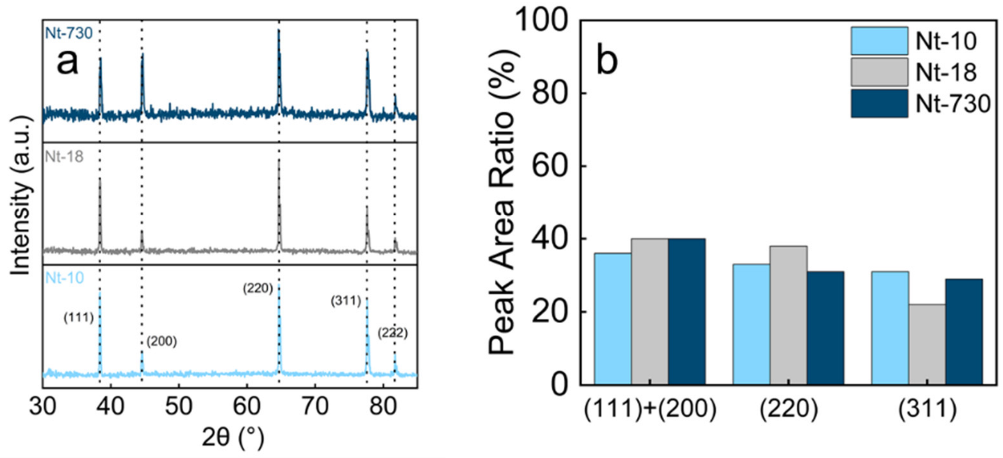

Supplementary Figure 4. Crystal facet characterization of silver samples. (**a**) The XRD pattern and (**b**) the facet percentage on Nt-10, Nt-18 and Nt-730.

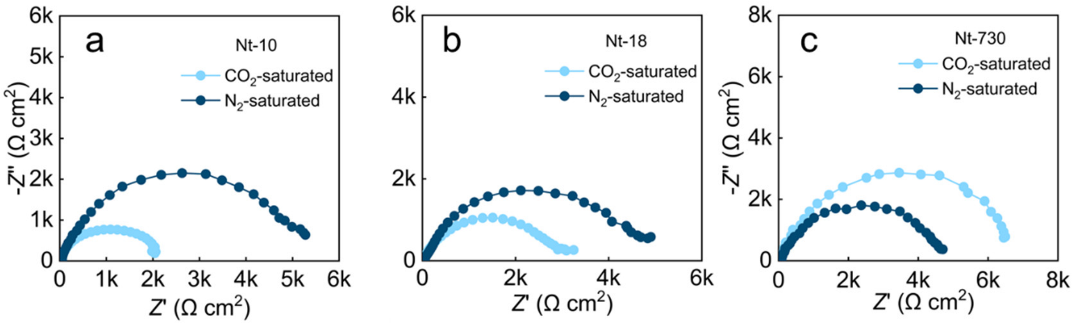

Supplementary Figure 5. Electrochemical impedance measurements. The Nyquist plots of (**a**) Nt-10, (**b**) Nt-18 and (**c**) Nt-730 in CO_2-saturated 0.5 M $KHCO_3$ solution.

Supplementary Table 2. Fitted charge-transfer resistance (R_{ct}) of Nt-10, Nt-18 and Nt-730 in CO_2-saturated and N_2-saturated solution

Samples	CO_2-saturated	N_2-saturated
Nt-10	2.0 kΩ cm^2	4.9 kΩ cm^2
Nt-18	2.5 kΩ cm^2	4.4 kΩ cm^2
Nt-730	6.3 kΩ cm^2	4.3 kΩ cm^2

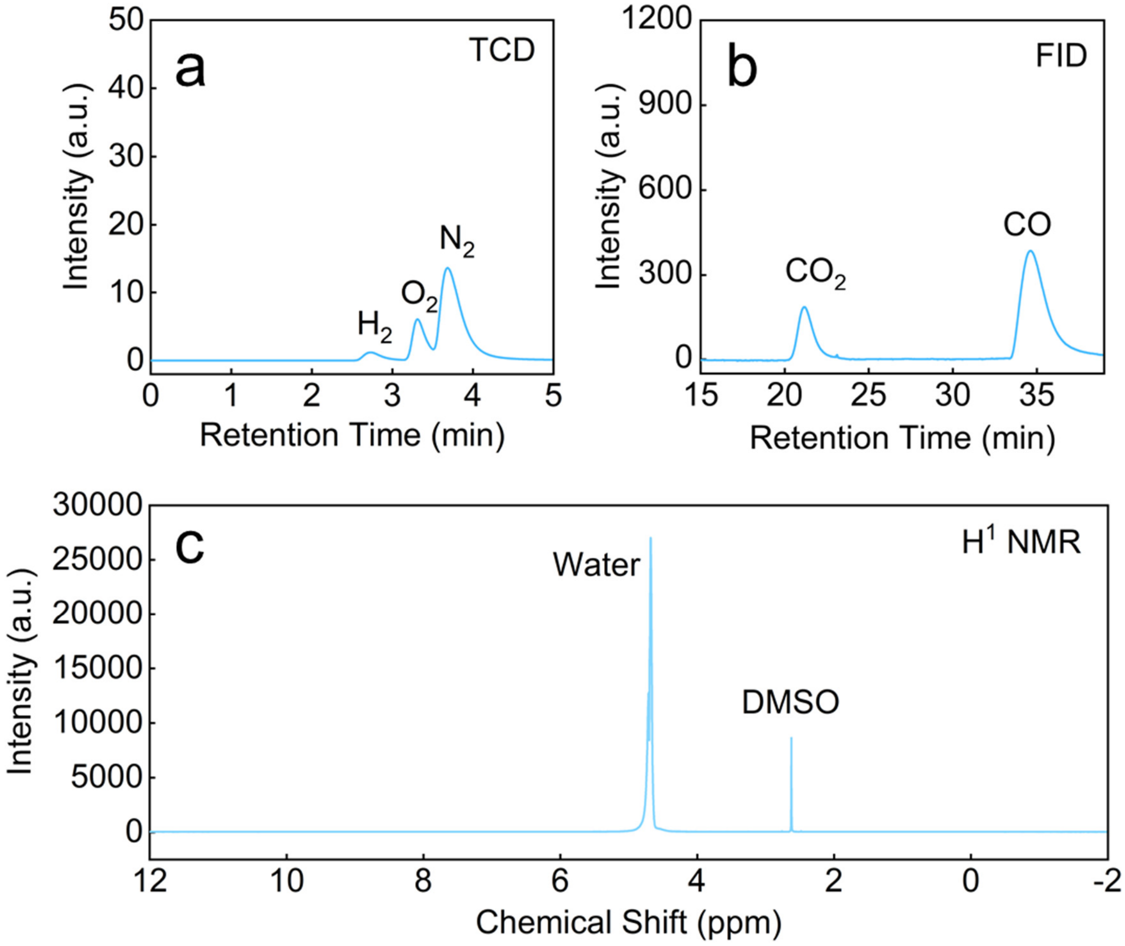

Supplementary Figure 6. Gaseous and liquid products detection after CO_2RR. (**a**) H_2 peak detected by TCD. (**b**) CO peak detected by FID. (**c**) ^{1}H NMR spectrum of electrolyte with DMSO as the internal standard. No liquid product was detected.

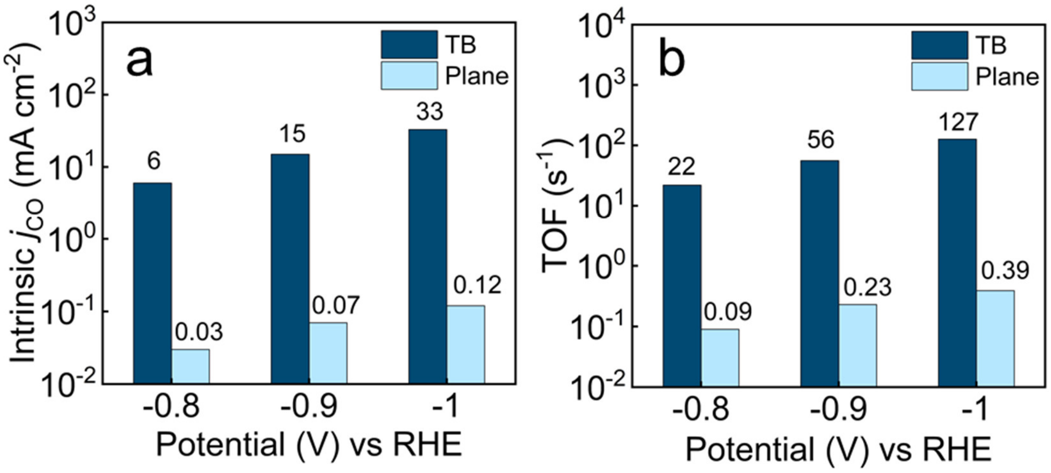

Supplementary Figure 7. Intrinsic electrocatalytic activity calculation. The (**a**) intrinsic j_{CO} and (**b**) TOF on TB and plane atoms.

Intrinsic Activity Calculation

The intrinsic activity calculation is based on three assumptions: i) The intrinsic activity is a constant value at a given potential and unvaried with TB density changing; ii) Mass transfer is efficient and doesn't control the reaction; iii) TB is the only active site besides facet atoms. The j_{CO} therefore linearly increases with TB density rising, and the slope in j_{CO}-TB density plot (k_{TB}) represents the j_{CO} per centimeter of TB. When TB density is zero, there is only plane atoms, indicating the interception is the intrinsic j_{CO} of plane (j_{plane}). This relationship can be expressed by the following equation:

$$j_{CO}(\text{mA cm}^{-2})=k_{TB}(\text{mA cm}^{-1})\times\text{TB density (cm}^{-1})+j_{plane}(\text{mA cm}^{-2}) \quad (3)$$

Assuming the area of TBs in electrode surface is TB density × silver's diameter (D_{Ag}, ~350 pm), the intrinsic j_{CO} of TB (j_{TB}) is as following:

$$j_{TB}(\text{mA cm}^{-2})=k_{TB}(\text{mA cm}^{-1})\div D_{Ag}(\text{cm}) \quad (4)$$

Turnover Frequency (TOF) Calculation

The TOF values of TB and plane atoms are based on their intrinsic j_{CO}. For TB atoms, the TOF of TB (TOF_{TB}) is calculated with equation (5):

$$TOF_{TB}=\frac{k_{TB}}{2\times F\times AD_{TB}} \quad (5)$$

Where F and AD_{TB} are faradaic constant and the amount of Ag atoms in one-centimeter-long TB, respectively.

In terms of plane atoms, we used {111} facet to replace the whole surface of the electrode. The atomic density of plane (AD_{plane}), which represents the amount of silver atoms in 1 cm^2 of {111} surface, is ~ 1.565×10^{-9} mol cm^{-2}. The TOF of plane atom (TOF_{plane}) is:

$$TOF_{plane}=\frac{j_{plane}}{2\times F\times AD_{plane}} \quad (6)$$

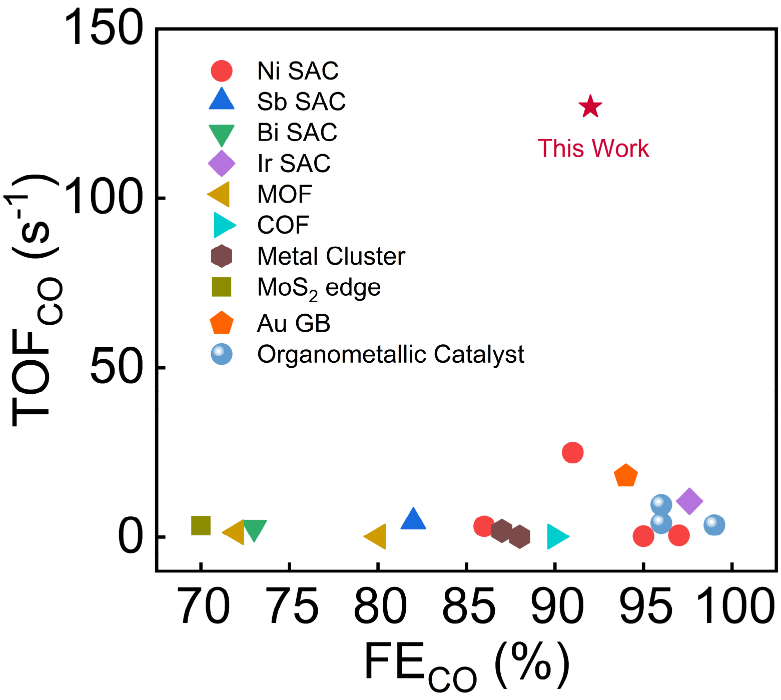

Supplementary Figure 8. The TOF_{CO} and FE_{CO} reported in Literatures 1-17. The detailed values are listed in Supplementary Table 3. (SAC: single-atom catalyst; MOF: metal-organic framework; COF: covalent organic framework; GB: grain boundary)

Supplementary Table 3. Literature summary about TOF_{CO} and FE_{CO}.

	TOF_{CO} (s^{-1})	FE_{CO} (%)	Reference
Silver's TB	127	92	This Work
Ni SAC	0.33	95	1
Ni SAC	0.55	97	2
Ni SAC	3.2	86	3
Ni SAC	25	91	4
Sb SAC	4.6	82	5
Bi SAC	2.8	73	6
Ir SAC	10.6	98	7
MOF	0.2	80	8
MOF	1.5	72	9
COF	0.21	90	10
Metal Cluster	0.17	88	11
Metal Cluster[a]	1.8	87	12
MoS_2 edge	3.5	70	13
Au GB[a]	18	94	14
Organometallic Catalyst	4.2	96	15
Organometallic Catalyst	9.6	96	16
Organometallic Catalyst	3.6	99	17

a. The TOF value is calculated by authors based on the literature data.

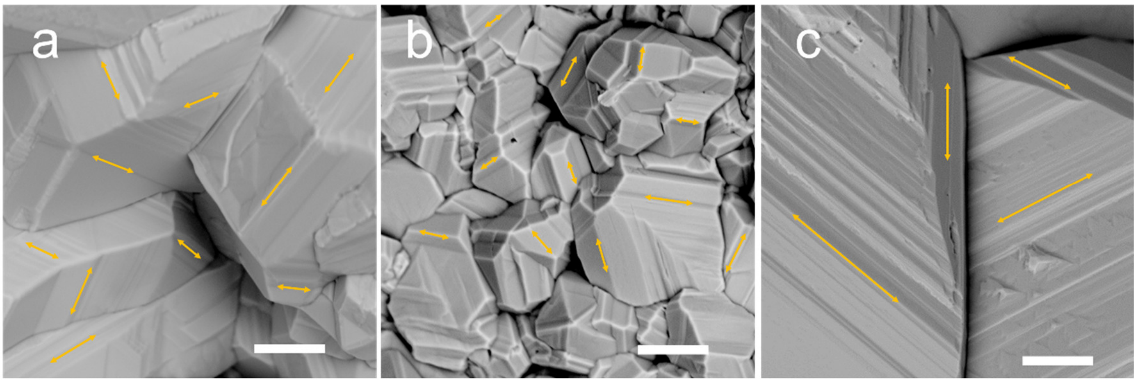

Supplementary Figure 9. Surface morphology of Nt-10 deposited on different substrates. SEM images of Nt-10 on **(a)** graphite paper, **(b)** silver foil and **(c)** carbon paper, respectively. The yellow arrows mark the TBs (scale bar: 5 μm).

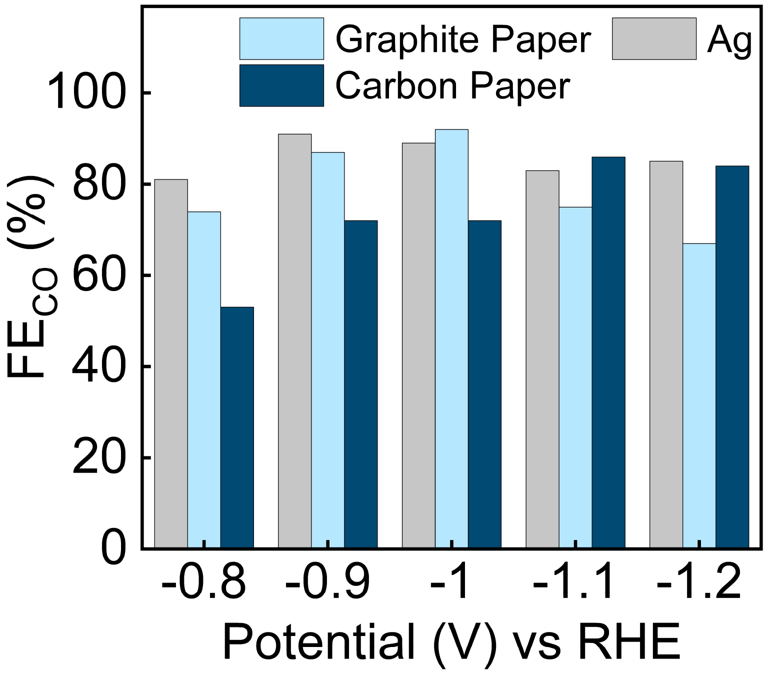

Supplementary Figure 10. The FE_{CO} of Nt-10 on different substrates.

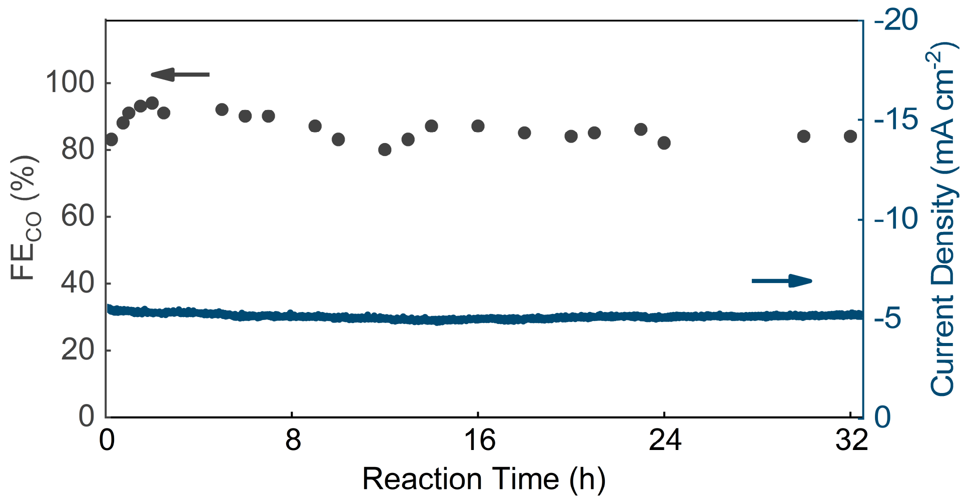

Supplementary Figure 11. Electrocatalytic stability of Nt-10. The current density and FE_{CO} of Nt-10 during a 32-hour reaction at -1.0 V.

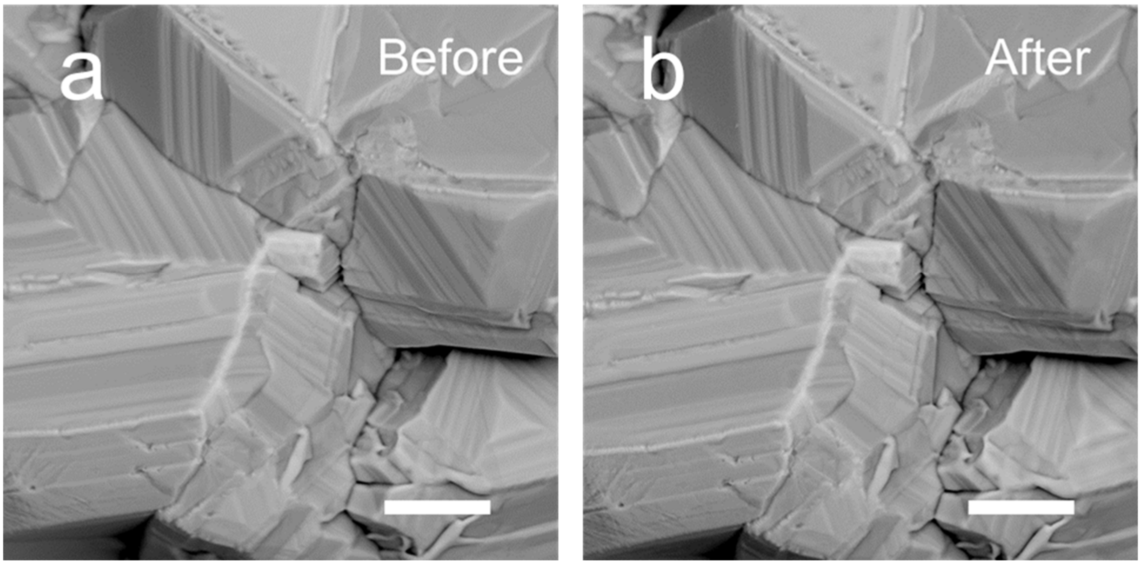

Supplementary Figure 12. Structural stability of Nt-10 in SEM observation. The SEM images of Nt-10 **(a)** before and **(b)** after 10-hour CO_2RR at -1.0 V (scale bar: 5 μm).

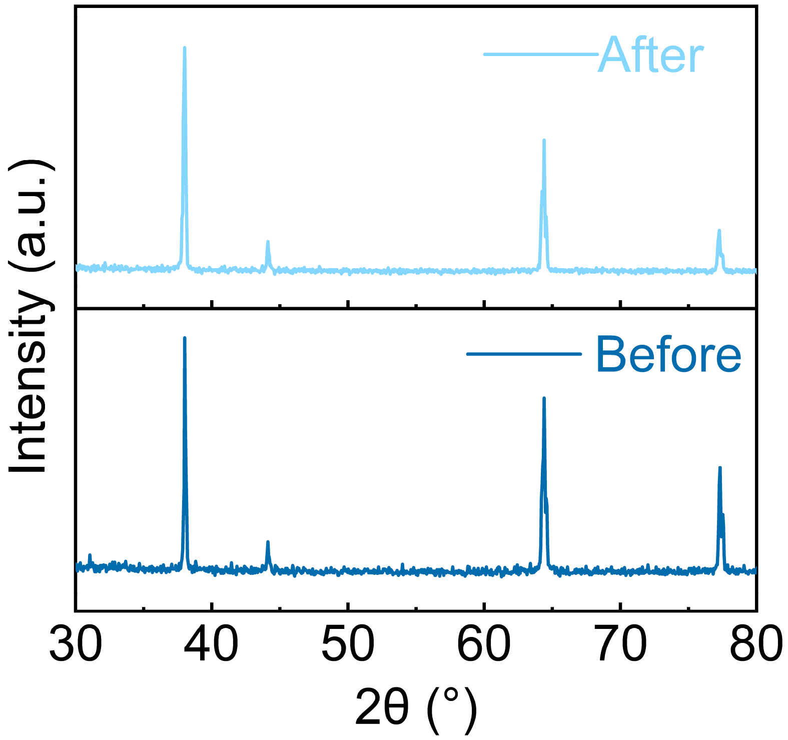

Supplementary Figure 13. Structural stability of Nt-10 in XRD measurement. The XRD patterns of Nt-10 before and after a 12-hour reaction at -1.0 V.

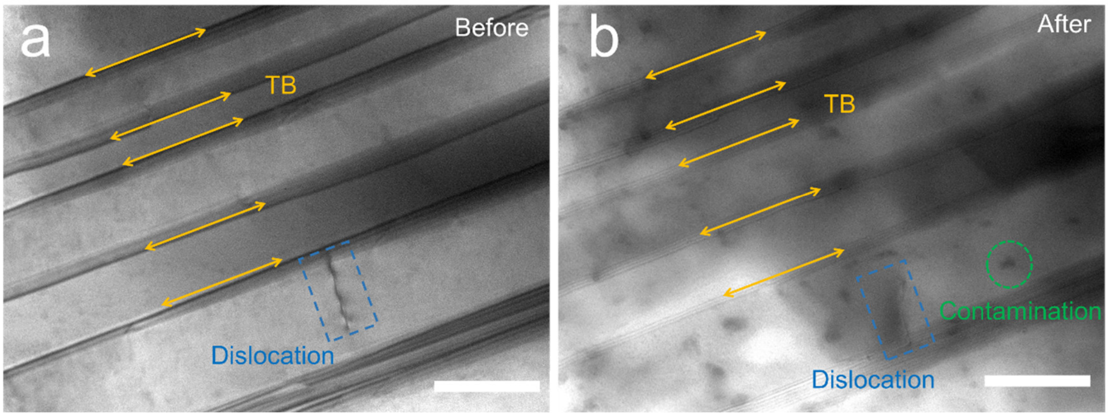

Supplementary Figure 14. Structural stability of Nt-10 in TEM observation. The TEM images of Nt-10 (**a**) before and (**b**) after a 12-hour reaction at -1.0 V (scale bar: 100 nm).

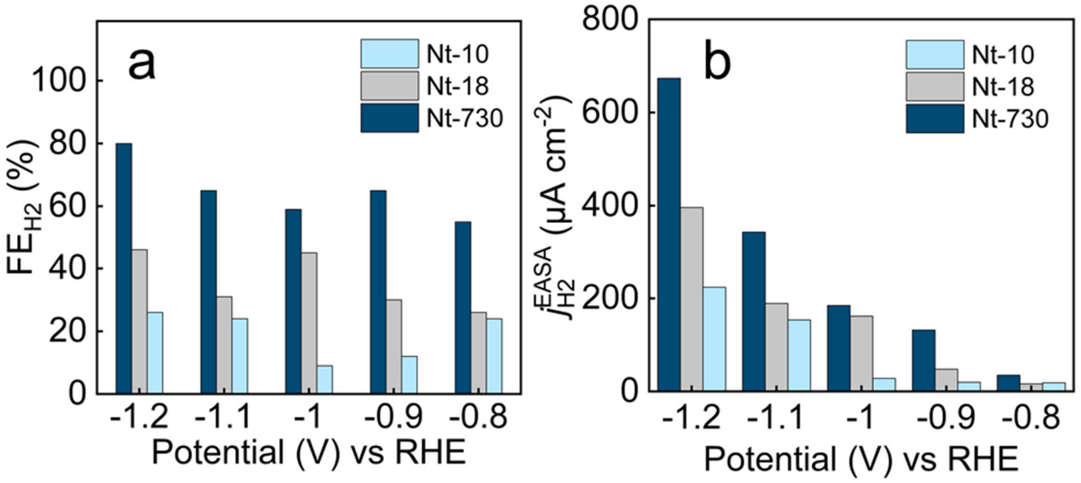

Supplementary Figure 15. Electrocatalytic performance of hydrogen evolution on silver catalysts. (**a**) FE_{H2} and (**b**) j_{H2}^{EASA} of Nt-10, Nt-18 and Nt-730 under different applied cathode potentials in CO_2-saturated solution.

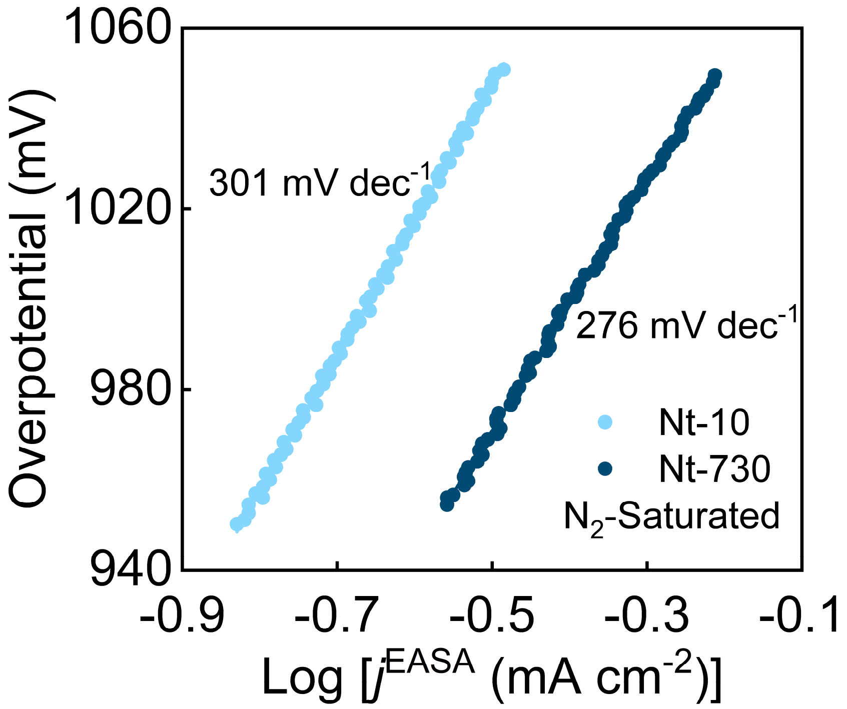

Supplementary Figure 16. Tafel plots in N_2-saturated solution on Nt-10 and Nt-730.

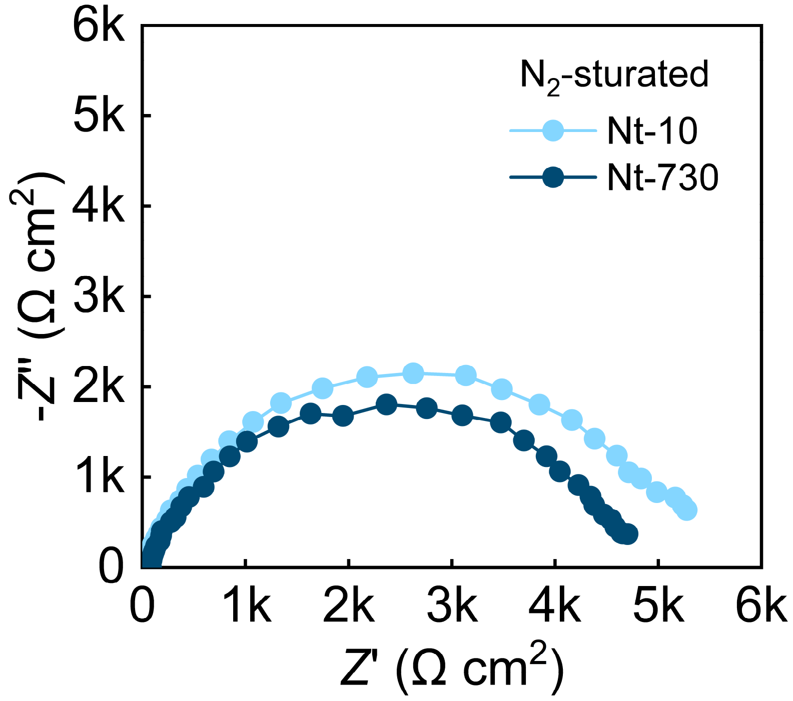

Supplementary Figure 17. Nyquist plots in N_2-saturated solution on Nt-10 and Nt-730.

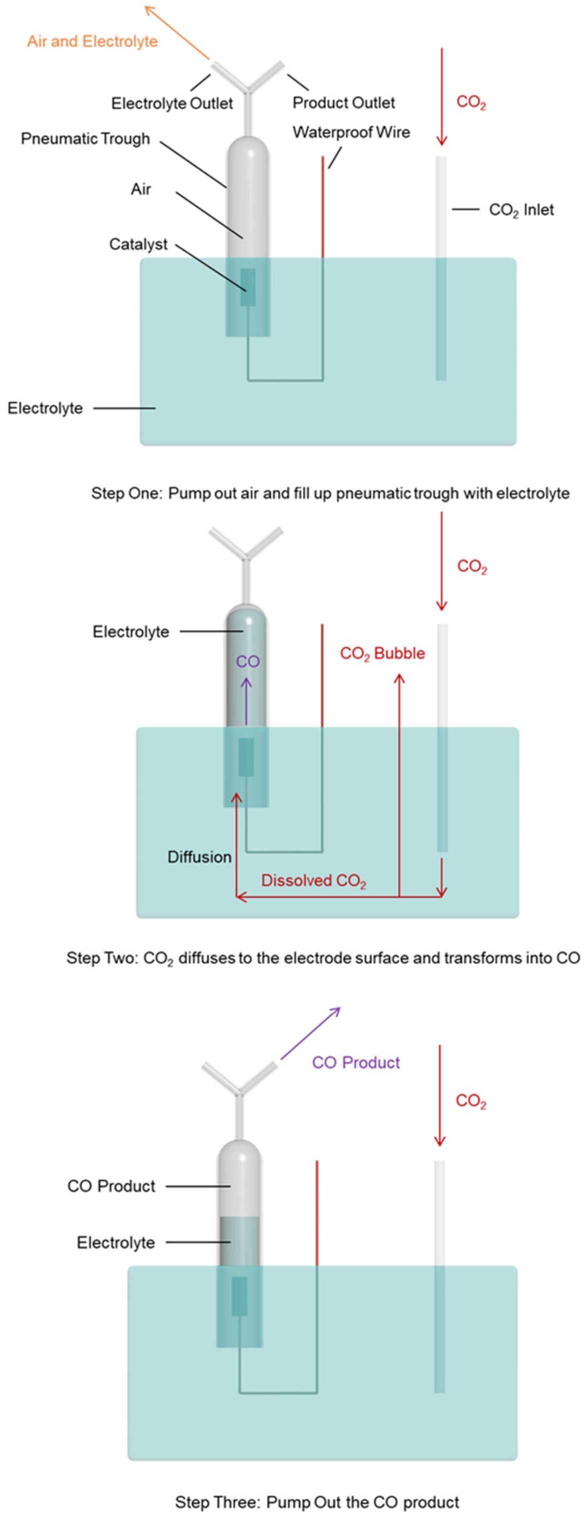

Supplementary Figure 18. Schematic diagram of pneumatic-trough-based electrochemical cell.

The electrode is connected with a waterproof wire and then inserted into pneumatic trough. Before the electrochemical reaction occurs, air in pneumatic trough is pumped out and electrolyte thereby fills in the pneumatic trough. CO_2 is bubbled from inlet, partially dissolves in the electrolyte and diffuses to the electrode surface to produce CO. The undissolved CO_2 is vented in the form of bubble outside the pneumatic trough. Therefore, the products we collected is relatively pure with some CO_2 diffusing from electrolyte. The CO product with high concentration is then pumped out for graphene growth.

Although the maximum volume fraction of CO can theoretically reach its FE_{CO}, the products can be practically diluted by CO_2 escaping from electrolyte. Therefore, the CO concentration is not only relative to the FE_{CO} but also controlled by CO_2 escape. In this system, there are two factors dominating CO_2 escape: temperature and surface roughness of device. Temperature affects partition coefficient of CO_2 between electrolyte and gas phase. The higher the temperature is, the larger the escape rate will be. In our experiment, the initial CO concentration can only reach up to ~33% at room temperature, but ~52% at 0 °C. A rough surface of device has more nucleate center for CO_2, which can accelerate CO_2 escape from electrolyte.

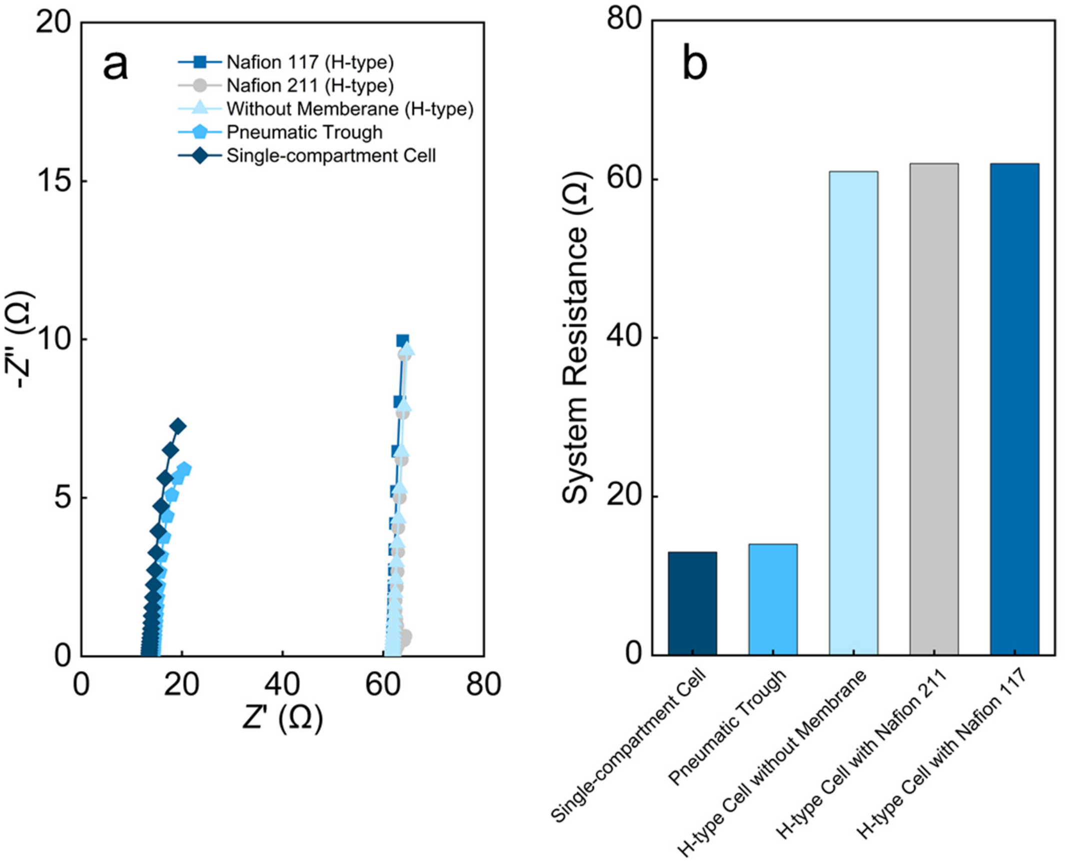

Supplementary Figure 19. Electrical impedance measurement of different systems. (**a**) System Impedance test in single-compartment cell, pneumatic-trough-based cell, H-type cell without membrane and H-type cell separated by Nafion 117 and Nafion 211 ion-exchange membrane. (**b**) The ohmic resistance measured from (**a**). The tests were performed at a potential of -2 V (vs. counter electrode) in 0.5 M $KHCO_3$ solution.

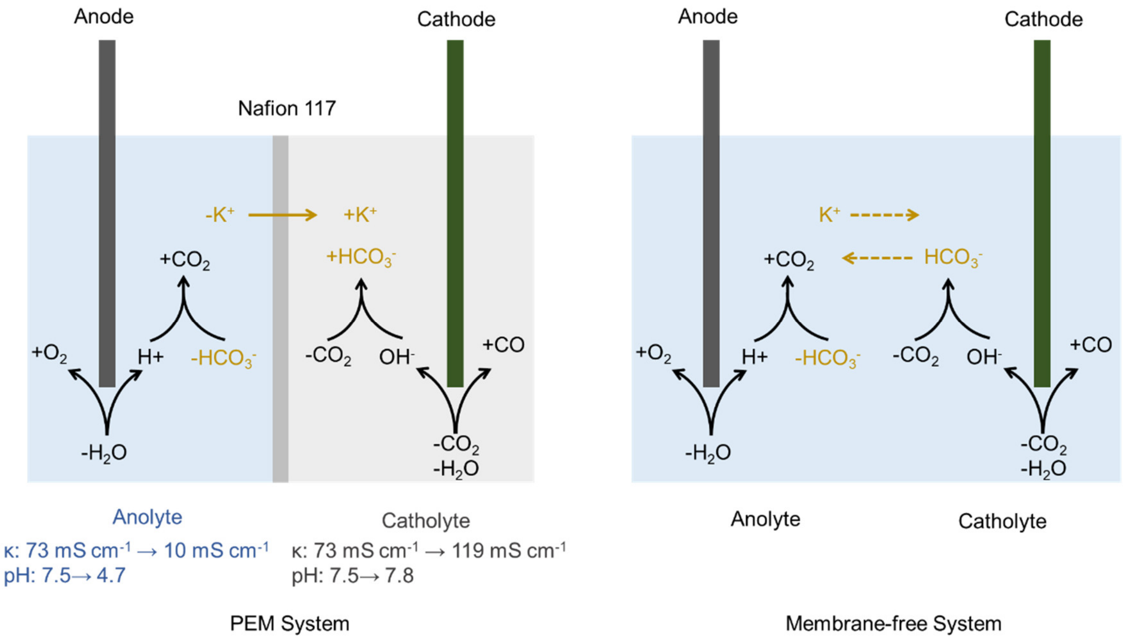

Supplementary Figure 20. The schematic of ion migration in proton-exchange membrane (PEM) system and membrane-free system.

In the bicarbonate-CO_2 electrolyte, the proton produced from OER quickly reacts with bicarbonate anion to generate CO_2 and water. The depletion of bicarbonate in the anolyte makes the potassium ions redundant, driving the K^+ ions to migrate across the membrane to the catholyte side. Meanwhile, the OH^- produced from CO_2RR reacts with CO_2 to form more bicarbonate in the catholyte, which cannot pass through the proton-exchange membrane, and balance with the influent K^+ ions in the catholyte. The overall effects give rise to a continuous depletion of $KHCO_3$ in the anolyte and a continuous accumulation of $KHCO_3$ in the catholyte.

To prove that, we monitored the pH value and electric conductivity (κ) changes in both anolyte and catholyte. The initial pH in both anolyte and catholyte is ~7.5, and the corresponding conductivity is ~ 73 mS cm^{-1}. After a 12-hour reaction, the pH and

conductivity in anolyte decreased to 4.7 and 10 mS cm^{-1}, respectively. Whereas, the pH and conductivity in catholyte rose to 7.8 and 119 mS cm^{-1}, respectively. This indicates both a $KHCO_3$ depletion in anolyte and a $KHCO_3$ accumulation in catholyte.

We think the “migration” of $KHCO_3$ is caused by the asymmetrical ion transfer in proton-exchange membrane, which can only admit the pass of cations. Without this membrane, we observed that H-type cell can maintain a stable cell voltage for much longer time (Fig. 3b), for the bicarbonate can diffuse freely and keep a constant concentration in both sides. We also measured the conductivity in membrane-free system, which shows little changes after running for 24 h.

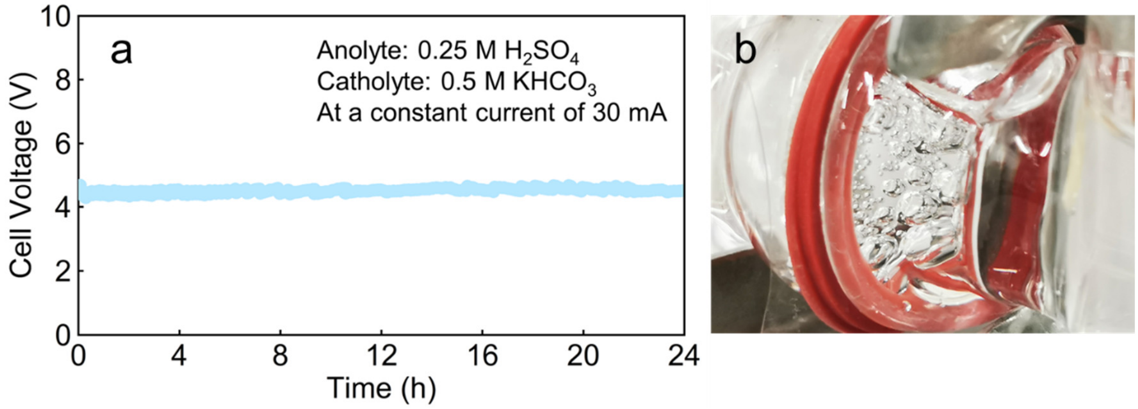

Supplementary Figure 21. Electrochemical measurement using sulfuric acid as anolyte in H-type cell. (**a**) The cell voltage of H-type cell separated by Nafion 117 membrane. The anolyte is 0.25 M H_2SO_4 and the catholyte is 0.5 M $KHCO_3$. (**b**) The corresponding optical image of the interface between Nafion 117 membrane and $KHCO_3$ catholyte.

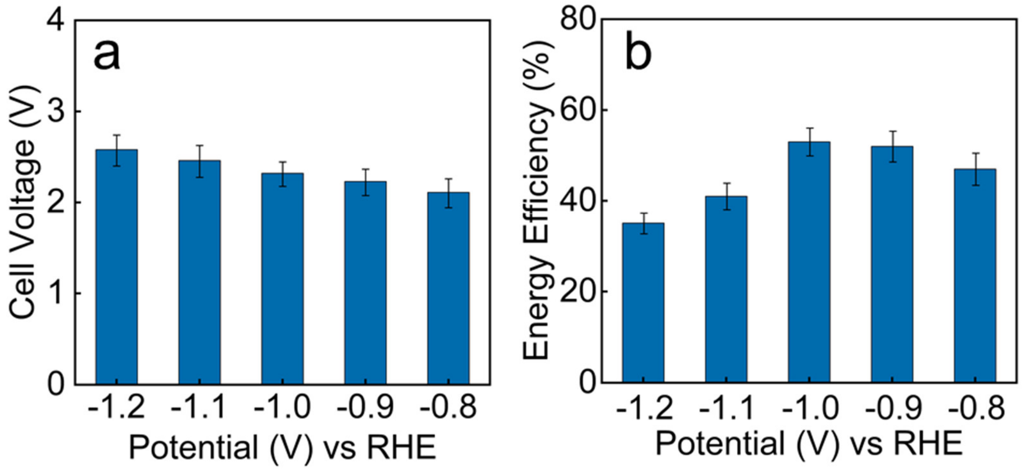

Supplementary Figure 22. Energy efficiency measurement and calculation. The (**a**) cell voltages and (**b**) energy conversion efficiencies at different potential on Nt-10 in a pneumatic-trough-based cell. The error bars represent the standard deviation, n = 3 independent replicates.

The energy conversion efficiency can be calculated by the following equation:

$$\text{Energy Efficiency}=\frac{(\Delta G_{\mathrm{m}}^{\theta}(\mathrm{CO})\text{-}\Delta G_{\mathrm{m}}^{\theta}(\mathrm{CO_2}))}{2UF}\times \mathrm{FE_{CO}} \quad (7)$$

Where $\Delta G_{\mathrm{m}}^{\theta}(\mathrm{CO})$ and $\Delta G_{\mathrm{m}}^{\theta}(\mathrm{CO_2})$ stand for the molar Gibbs free energy of CO and CO_2, equaling to -137.168 kJ mol^{-1} and -394.359 kJ mol^{-1}, respectively. U is the absolute value of cell voltage and F is Faradaic constant.

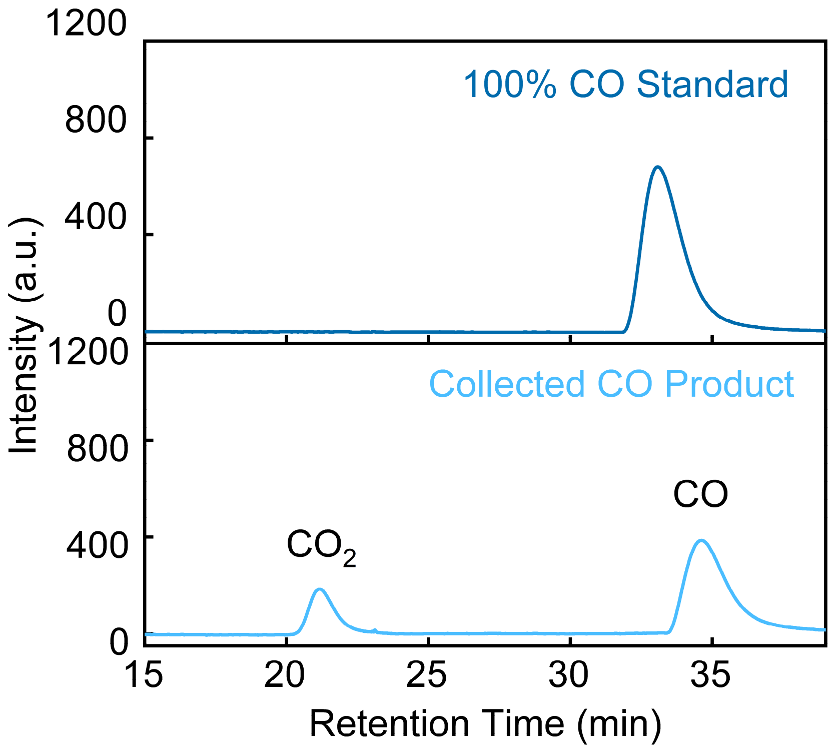

Supplementary Figure 23. CO concentration measurement. The GC spectrum of CO standard and collected CO product under the most optimal condition.

Supplementary Table 4. Literature summary about FE_{CO} and initial CO concentration.

Electrocatalyst	Reaction System	FE_{CO} (%)	CO Concentration (%)	Reference
Nt-Ag	Pneumatic Trough	92	52	This work
NiPc/CNT	GDE	99.5	12	18
Ni@NCH	GDE	90	2.3	19
Ni-NG	GDE	97	3.0	20
Zn	GDE	84	1.3	21
Ag/C	GDE	90	2.8	22
Ag	GDE	90	4.2	23
Au/MWCNT	GDE	90	4.3	24
NiN-GS	GDE	90	10	25
Ag NPs	GDE	70	4.4	26
Ag NPs	GDE	96	24	27
Ag NPs	GDE	58	49	28
Cu/In	H-type	80	2.0	29
Ni-NG	H-type	97	0.23	20
Ni-NSG	H-type	97	0.76	30
Zn	H-type	94	0.38	21
$Co-N_2$	H-type	94	1.3	31
Li-Zn	H-type	91	2.0	32
Ag_2O-ZIF	H-type	80	0.82	33
Ag/C	H-type	86	0.028	34
NiN-GS	H-type	93	0.28	25
$Co-N_5$	H-type	99	0.039	35
Ag NPs	Carbonate Reduction	25	25	36
Ag NPs	Bicarbonate Reduction	62	41	37

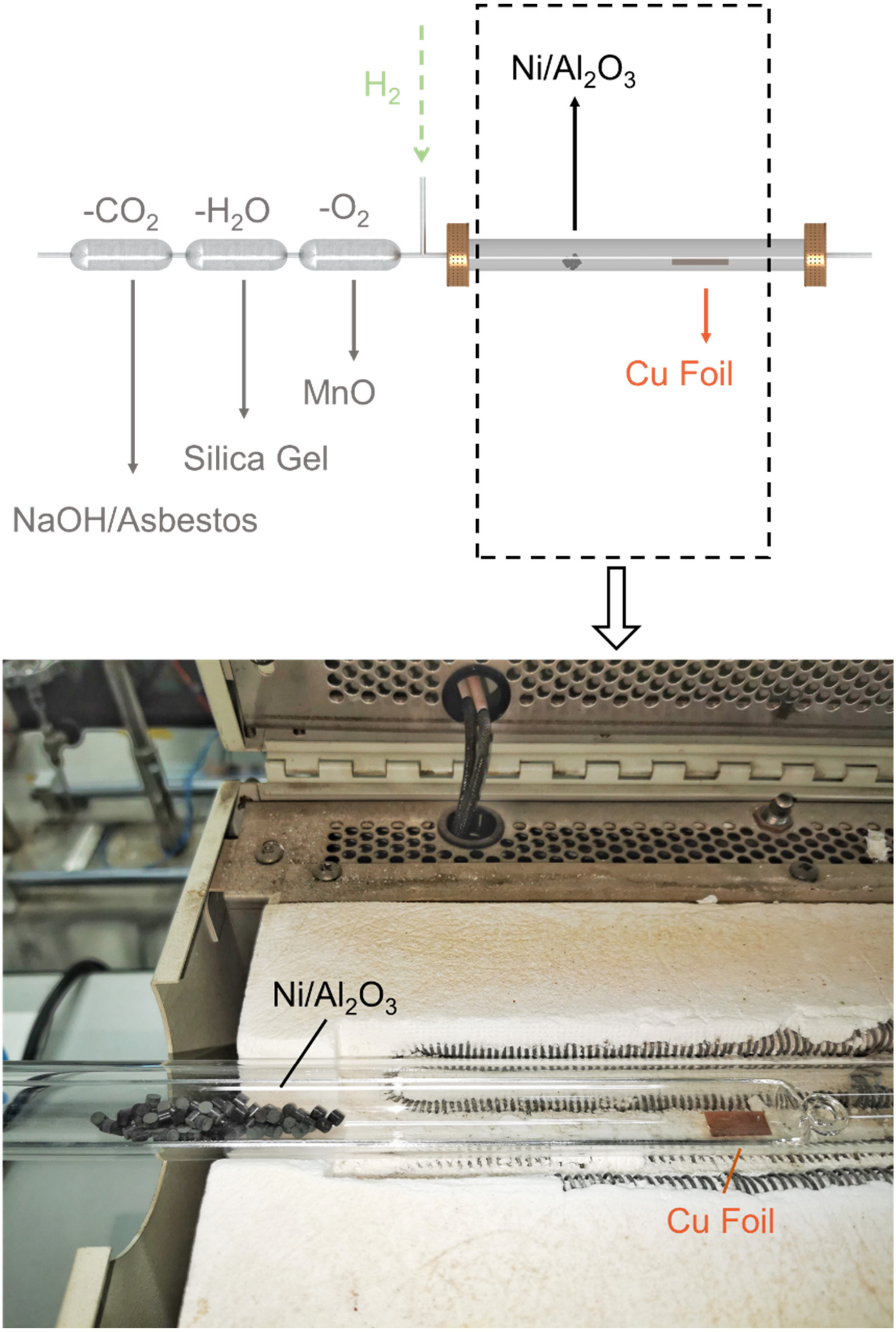

Supplementary Figure 24. Graphene growth system. The schematic setup and photo of graphene growth system.

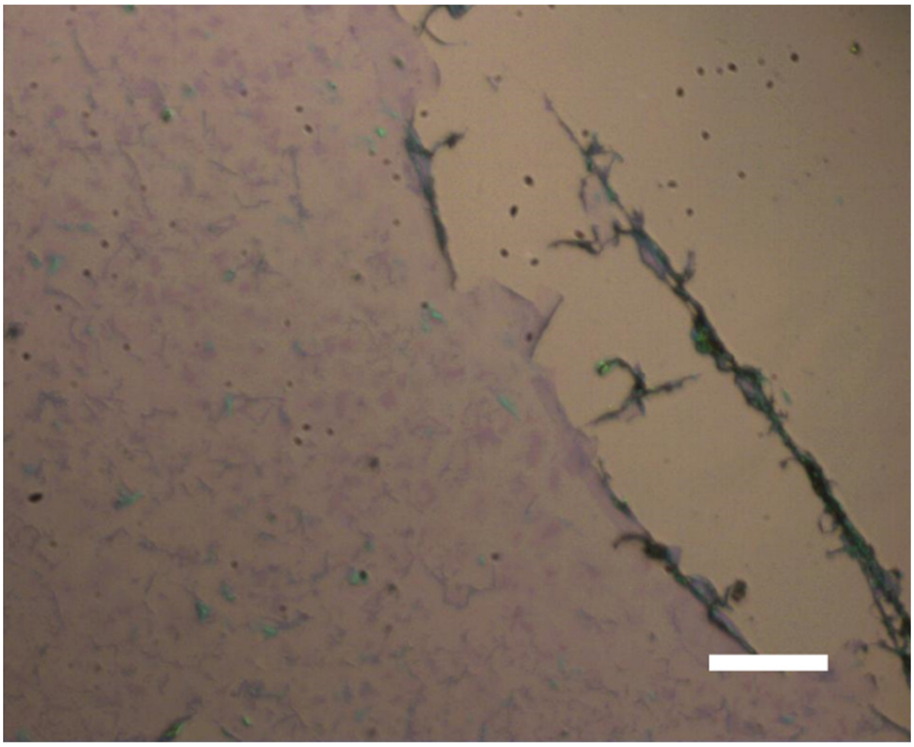

Supplementary Figure 25. Optical image of graphene film on SiO_2 substrate synthesized from CO product. The scale bar represents 50 μm.

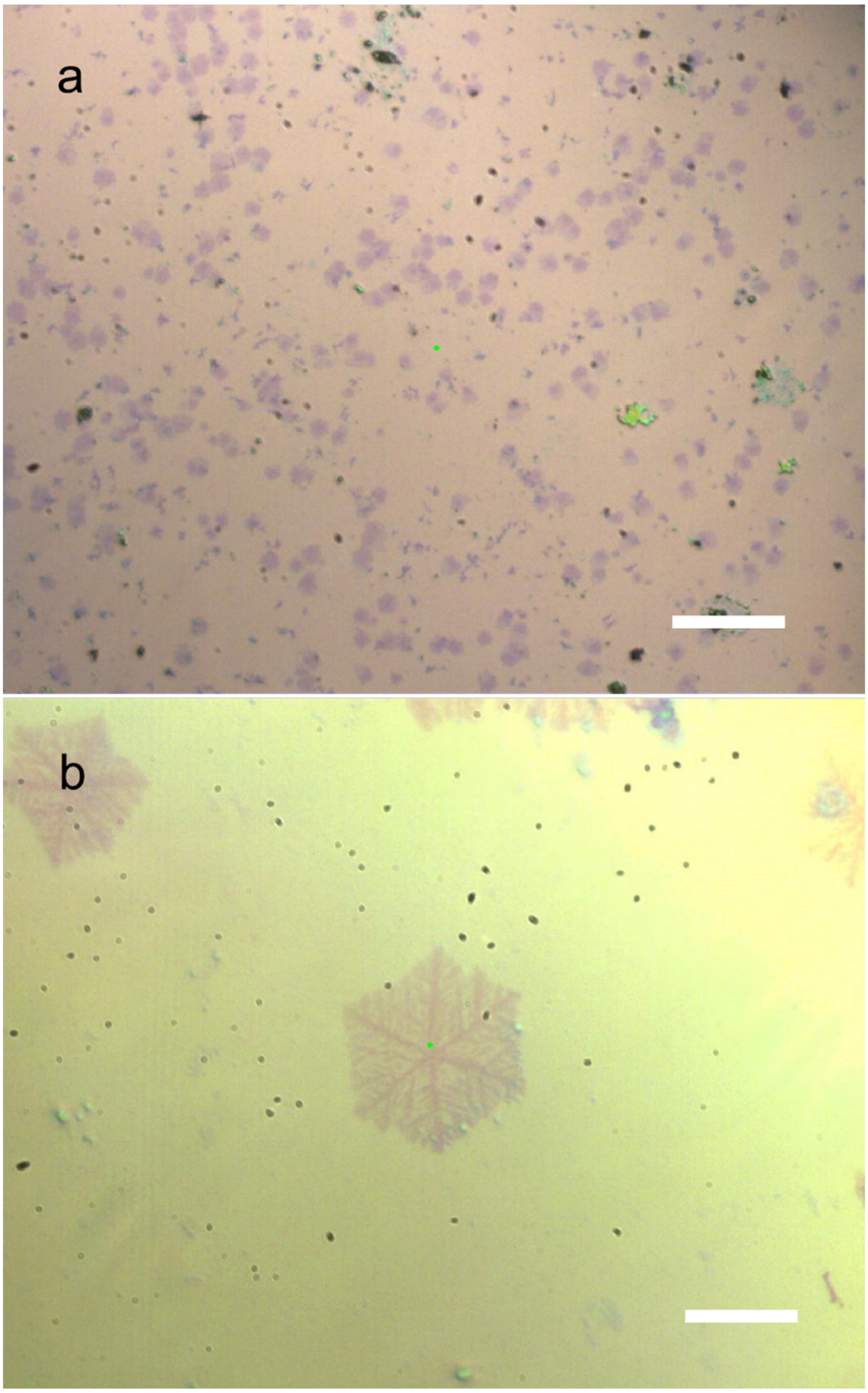

Supplementary Figure 26. Optical image of graphene fragment on SiO_2 substrate synthesized from pure CO_2. (a) scale bar: 100 μm; (b) scale bar: 10 μm.

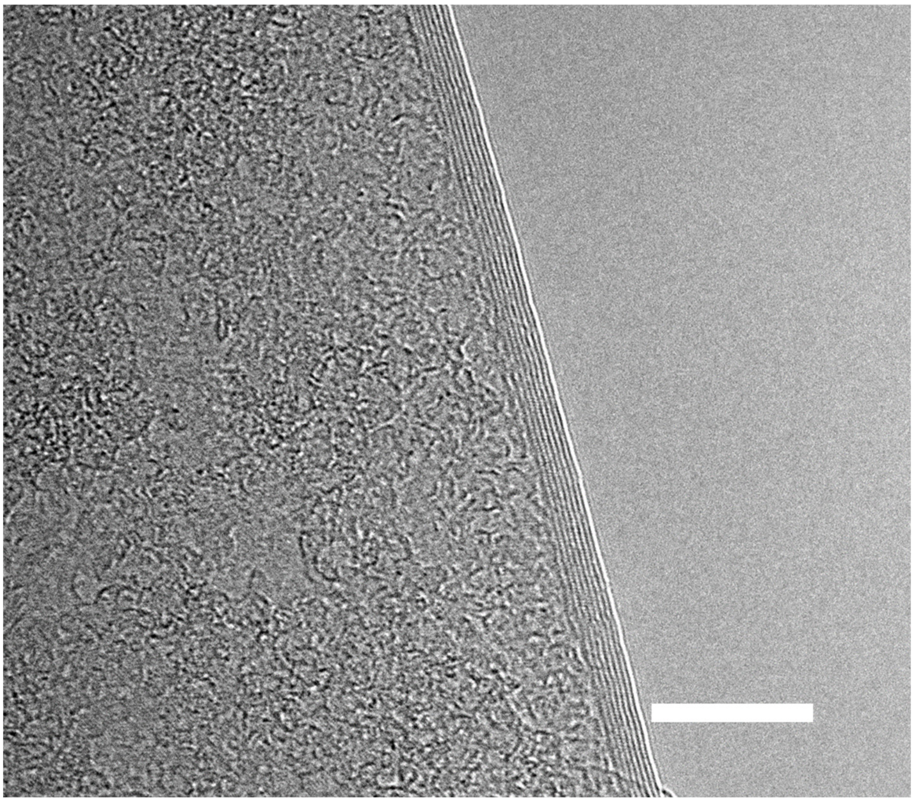

Supplementary Figure 27. TEM images of graphene fragments from pure CO_2.

The scale bar represents 10 nm.

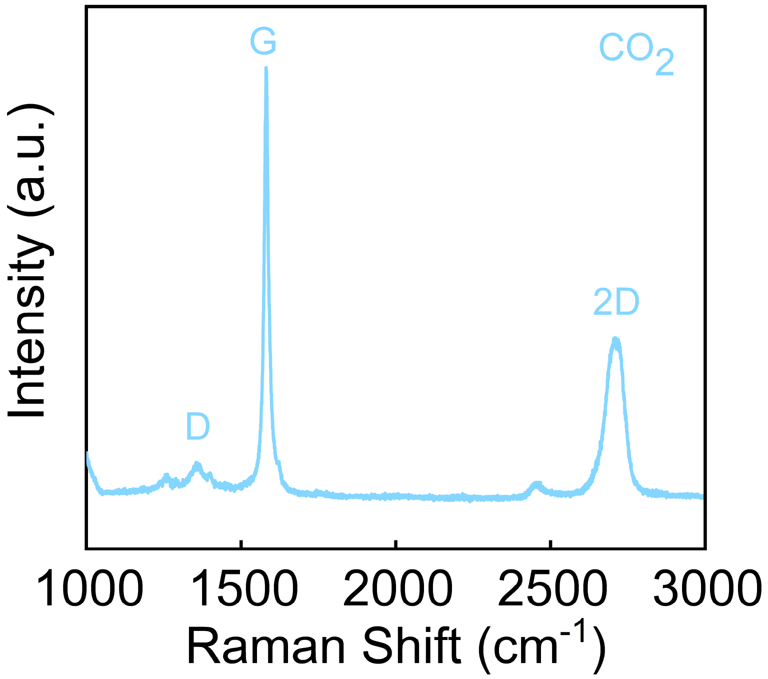

Supplementary Figure 28. Raman spectrum of graphene fragments synthesized from pure CO_2.

References:

1. Lu, P. et al. Facile synthesis of single-nickel-atomic dispersed N-doped carbon framework for efficient electrochemical CO_2 reduction. *Appl. Catal. B: Environ.* **241**, 113-119 (2019).

2. Zhang, T. et al. Atomically dispersed nickel (I) on an alloy-encapsulated nitrogen-doped carbon nanotube array for high-performance electrochemical CO_2 reduction reaction. *Angew. Chem.* **132**, 12153-12159 (2020).

3. Fan, Q. et al. Activation of Ni particles into single Ni–N atoms for efficient electrochemical reduction of CO_2. *Adv. Energy Mater* **10**, 1903068 (2020).

4. Cheng, Y. et al. Atomically dispersed transition metals on carbon nanotubes with ultrahigh loading for selective electrochemical carbon dioxide reduction. *Adv. Mater.* **30**, 1706287 (2018).

5. Jia, M., Hong, S., Wu, T.-S., Soo, Y.-L. & Sun, Z.; Single Sb sites for efficient electrochemical CO_2 reduction. *Chem. Commun.* **55**, 12024-12027 (2019).

6. Zhang, E. et al. Bismuth single atoms resulting from transformation of metal–organic frameworks and their use as electrocatalysts for CO_2 reduction. *J. Am. Chem. Soc.* **141**, 16569-16573 (2019).

7. Sun, X. et al. Aqueous CO_2 reduction with high efficiency using α-$Co(OH)_2$-supported atomic Ir electrocatalysts. *Angew. Chem. Int. Edit.* **58**, 4669-4673 (2019).

8. Matheu, R. et al. Three-dimensional phthalocyanine metal-catecholates for high electrochemical carbon dioxide reduction. *J. Am. Chem. Soc.* **141**, 17081-17085 (2019).

9. Zhao, C. et al. Ionic exchange of metal–organic frameworks to access single nickel

sites for efficient electroreduction of CO_2. *J. Am. Chem. Soc.* **139**, 8078-8081 (2017).

10. Lin, S. et al. Covalent organic frameworks comprising cobalt porphyrins for catalytic CO_2 reduction in water. *Science* **349**, 1208-1213 (2015).

11. Gao, D., Zhou, H., Wang, J., Wang, G. & Bao, X. Pd-containing nanostructures for electrochemical CO_2 reduction reaction. *ACS Catal.* **8**, 1510-1519 (2018).

12. Kauffman, D. R. et al. Efficient electrochemical CO_2 conversion powered by renewable energy. *ACS Appl. Mater Interfaces* **7**, 15626-15632 (2017).

13. Abbasi, P. et al. Tailoring the edge structure of molybdenum disulfide toward electrocatalytic reduction of carbon dioxide. *ACS nano* **11**, 453-460 (2016).

14. Feng, X., Jiang, K., Fan, S. & Kanan, M. W. Grain-boundary-dependent CO_2 electroreduction activity. *J. Am. Chem. Soc.* **137**, 4606-4609 (2015).

15. Han, J. et al. Reordering d orbital energies of single-site catalysts for CO_2 electroreduction. *Angew. Chem. Int. Edit.* **58**, 12711-12716 (2019).

16. Sun, L. et al. A planar, conjugated N_4-macrocyclic cobalt complex for heterogeneous electrocatalytic CO_2 reduction with high activity. *Angew. Chem.* **132**, 2-8 (2020).

17. Ma, D.-D. et al. Remarkable electrocatalytic CO_2 reduction with ultrahigh CO/H_2 ratio over single-molecularly immobilized pyrrolidinonyl nickel phthalocyanine. *Appl. Catal. B: Environ.* **264**, 118530 (2020).

18. Zhang, X. et al. Molecular engineering of dispersed nickel phthalocyanines on carbon nanotubes for selective CO_2 reduction. *Nat. Energy* **5**, 684-692 (2020).

19. Daiyan, R. et al. Transforming active sites in nickel–nitrogen–carbon catalysts for

efficient electrochemical CO_2 reduction to CO. *Nano Energy* **78**, 105213 (2020).

20. Jiang, K. et al. Isolated Ni single atoms in graphene nanosheets for high-performance CO_2 reduction. *Energ. Environ. Sci.* **11**, 893-903 (2018).

21. Luo, W., Zhang, J., Li, M. & Züttel, A. Boosting CO production in electrocatalytic CO_2 reduction on highly porous Zn catalysts. *ACS Catal.* **9**, 3783-3791 (2019).

22. Dinh, C.-T., García de Arquer, F. P., Sinton, D.& Sargent, E. H. High rate, selective, and stable electroreduction of CO_2 to CO in basic and neutral media. *ACS Energy Lett.* **3**, 2835-2840 (2018).

23. Haas, T., Krause, R., Weber, R., Demler, M. & Schmid, G. Technical photosynthesis involving CO_2 electrolysis and fermentation. *Nat. Catal.* **1**, 32-39 (2018).

24. Verma, S. et al. Insights into the low overpotential electroreduction of CO_2 to CO on a supported gold catalyst in an alkaline flow electrolyzer. *ACS Energy Lett.* **3**, 193-198 (2017).

25. Jiang, K. et al. Transition-metal single atoms in a graphene shell as active centers for highly efficient artificial photosynthesis. *Chem* **3**, 950-960 (2017).

26. Salvatore, D. A. et al. Electrolysis of gaseous CO_2 to CO in a flow cell with a bipolar membrane. *ACS Energy Lett.* **3**, 149-154 (2017).

27. Romero Cuellar, N. S. et al. Two-step electrochemical reduction of CO_2 towards multi-carbon products at high current densities. *J. CO_2 Util.* **36**, 263-275 (2020).

28. Krause, R. et al. Industrial application aspects of the electrochemical reduction of CO_2 to CO in aqueous electrolyte. *Chem. Ing. Tech.* **92**, 53-61, (2020).

29. Luo, W. et al. Selective and stable electroreduction of CO_2 to CO at the

copper/indium interface. *ACS Catal.* **8**, 6571-6581 (2018).

301. Yang, H. B. et al. Atomically dispersed Ni (I) as the active site for electrochemical CO_2 reduction. *Nat. Energy* **3**, 140-147 (2018).

31. Wang, X. et al. Regulation of coordination number over single Co sites: triggering the efficient electroreduction of CO_2. *Angew. Chem.* **130**, 1962-1966 (2018).

32. Jiang, K., Wang, H., Cai, W.- B. & Wang, H. Li electrochemical tuning of metal oxide for highly selective CO_2 reduction. *ACS nano* **11**, 6451-6458 (2017).

33. Jiang, X. et al. Boosting CO_2 electroreduction over layered zeolitic imidazolate frameworks decorated with Ag_2O nanoparticles. *J. Mater. Chem. A* **5**, 19371-19377 (2017).

34. Gao, Y. et al. Enhanced selectivity and activity for electrocatalytic reduction of CO_2 to CO on an anodized Zn/carbon/Ag electrode. *J. Mater. Chem. A* **7**, 16685-16689 (2019).

35. Pan, Y. et al. Design of single-atom Co–N_5 catalytic site: a robust electrocatalyst for CO_2 reduction with nearly 100% CO selectivity and remarkable stability. *J. Am. Chem. Soc.* **140**, 4218-4221 (2018).

36. Li, Y. C. et al. CO_2 electroreduction from carbonate electrolyte. *ACS Energy Lett.* **4**, 1427-1431 (2019).

37. Lees, E. W. et al. Electrodes designed for converting bicarbonate into CO. *ACS Energy Lett.* **5**, 2165-2173 (2020).
